# Enhanced Antibacterial Efficacy of Copper Single-Atom Catalysts on a Two-Dimensional Boron Nitride Platform

**DOI:** 10.1021/acsnano.5c13145

**Published:** 2025-12-10

**Authors:** Wenbo Li, Daniel Maldonado-Lopez, Yingcan Zhao, Cong Wang, Jianxiang Gao, Bowen Sun, Yichao Bai, Linxuan Sun, Mingchuang Zhao, Haoqi He, Jiatao Lou, Qiangmin Yu, Xi Zhang, Vijay Kumar Pandey, Feiyu Kang, Mauricio Terrones, Jose L. Mendoza-Cortes, Yu Lei

**Affiliations:** † Institute of Materials Research, Center of Double Helix, Tsinghua Shenzhen International Graduate School, 118351 Tsinghua University, Shenzhen 518055, P. R. China; ‡ Shenzhen Key Laboratory of Advanced Layered Materials for Value-Added Applications, Institute of Materials Research, Tsinghua Shenzhen International Graduate School, 12442 Tsinghua University, Shenzhen 518055, P. R. China; § Department of Chemical Engineering & Materials Science, 3078 Michigan State University, East Lansing, Michigan 48824, United States; ∥ Environmental Science Program, Department of Life Sciences, 125809 Beijing Normal University-Hong Kong Baptist University United International College, Guangdong 519087, P. R. China; ⊥ Institute of Biopharmaceutical and Health Engineering, Tsinghua Shenzhen International Graduate School, 551667 Tsinghua University, Shenzhen 518055, P. R. China; # Shenzhen Geim Graphene Center, Tsinghua-Berkeley Shenzhen Institute & Shenzhen International Graduate School, Tsinghua University, Shenzhen 518055, P. R. China; ∇ Department of Physics, Department of Chemistry, Department of Materials Science and Engineering, Center for Two-Dimensional and Layered Materials, 311285 The Pennsylvania State University, University Park, Pennsylvania 16802, United States; ○ Department of Physics & Astronomy, Michigan State University, East Lansing, Michigan 48824, United States

**Keywords:** 2D materials, boron nitride, Cu single-atom catalysts, photocatalysis, reactive oxygen species, antibacterial

## Abstract

Copper-based antibacterial systems leverage reactive oxygen species (ROS) for effective pathogen control but are limited by issues such as cytotoxicity and resistance due to Cu ion release. By anchoring copper single-atom catalysts (Cu SACs) on biocompatible boron nitride (BN) nanosheets, we create a stable, high-efficiency antibacterial platform that minimizes Cu-ion-induced cytotoxicity and bacterial resistance. This configuration maximizes metal utilization and enhances photocatalytic efficiency for ROS generation, including hydroxyl radicals and superoxide anions. The defect-assisted covalent bonding between Cu and BN ensures stable coordination, preventing metal ion dissolution. First-principles quantum calculations at the level of density functional theory (DFT) provided critical insights into the structures and mechanisms of ROS generation, showing how atomic-level interactions between Cu and BN surfaces boost catalytic activity and clarified electron transfer processes and adsorption energies essential for ROS formation. These insights explain the observed catalytic behaviors and provide valuable design principles for developing efficient, low-toxicity SAC-based antibacterial systems. Additionally, we studied other elements in the same row (Cr, Mn, Fe, Co, Ni, and Zn) experimentally and theoretically. The d-BN-Cu system rapidly inactivated *E. coli* (10^6^ CFU mL^–1^), achieving significant results with d-BN-Cu_1_ (Cu, 0.26 at. % with Cu nanoclusters) within 15 min, and d-BN-Cu_3_ (Cu, 0.024 at. % with Cu SAC) within 30 min when exposed to sunlight. Although higher copper content can achieve better antibacterial effects, it also brings other potential risks, such as metal ion leaching and higher cytotoxicity. This risk can be effectively avoided by utilizing SACs, as all of the Cu SACs are securely anchored at the defect sites in h-BN through covalent bonds. Cell toxicity testing and *in vivo* testing emphasize the unique advantages of d-BN-Cu_3_ (SAC) in balancing safety and efficiency. This SAC two-dimensional platform can not only effectively combat Gram-negative and Gram-positive bacteria but also effectively avoid the toxicity caused by the metal itself.

## Graphical abstract

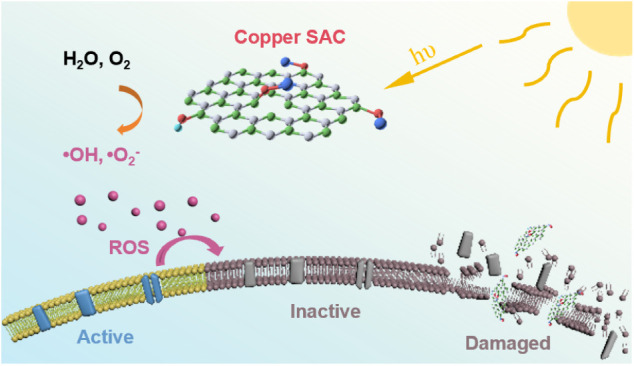

## Introduction

The discovery of antibiotics has greatly improved public health conditions and is widely used in humans and animals.^1^ However, the overuse of antibiotics has led to the emergence of antimicrobial resistance (AMR) in bacteria, posing a serious global threat to public health and presenting one of the most pressing challenges of our time.^2–4^ Reactive oxygen species (ROS), a primary antibacterial mechanism, can destroy bacterial cell membranes, proteins, DNA, *etc*., thereby killing bacteria without introducing AMR.^5–8^ Therefore, copper (Cu)-based antimicrobial systems with high ROS catalytic activity have received considerable attention in recent years.^9–12^ However, the tendency of metal nanoparticles to agglomerate during catalytic processes significantly reduces their efficiency, while the release of Cu ions poses additional environmental concerns.^13–16^ Single-atom catalysts (SACs) show promise for antibacterial systems by enhancing atomic utilization and minimizing toxic ion release, which is crucial for sustainable pathogen control. SACs can enhance the density of active sites, improve atomic utilization, and maximize the catalytic properties of metals.^17–19^ Moreover, the aggregation of metallic elements can be physically hindered by anchoring the metal atoms to the substrates.^20–22^ Meanwhile, the local coordination environment of the metal single atoms is also a key parameter to regulate the catalytic performance,^23^ making the selection and coordination of SAC substrates particularly important.

Two-dimensional (2D) materials exhibit two main antibacterial mechanisms: physical puncturing of bacterial cell membranes enabled by their layered structure,^24^ and bacterial inactivation through oxidative stress generated by electron transfer reactions.^25^ These mechanisms have established 2D materials as emerging antibacterial agents. At the same time, their flat structure provides a larger specific surface area and active sites, making them a good carrier for antibacterial materials.^26,27^ Among the 2D materials family, boron nitride (BN) has excellent physical and chemical properties, such as high-temperature resistance, high strength, and corrosion resistance,^28^ and also exhibits excellent performance and biocompatibility in the antibacterial field.^29^ It has been widely used in antibacterial, antitumor, and drug delivery fields.^16,30,31^ Despite these promising applications, the antimicrobial performance of BN still has much room for improvement compared with conventional organic and metallic antimicrobial materials.

Herein, we prepared BN nanosheets loaded with Cu SACs (d-BN-Cu_3_) and used density functional theory (DFT) to identify the optimal coordination form of Cu SACs (B–O–Cu) for ROS catalysis. First, defective BN (d-BN) was obtained by cryomilling, which reduced the BN’s lateral size and thickness, increased its specific surface area, and introduced more surface defects.^32^ The smaller BN size after cryomilling enhances its ability to physically disrupt bacterial cells, thereby improving its antibacterial performance.^33^ Additionally, the increased specific surface area and abundance of functional groups (−OH) introduced as surface defects provide more sites for oxidative stress activation and optimal loading of SACs. This approach enables the Cu-loaded BN nanosheets to function as a synergistic bactericidal agent, with Cu SACs on the surface enhancing antibacterial activity. The structure of Cu SACs was verified by aberration-corrected scanning transmission electron microscopy (STEM) and extended X-ray absorption fine structure (EXAFS) analysis. The ROS catalytic ability of the materials was verified by electron paramagnetic resonance (EPR) testing, demonstrating that d-BN-Cu_3_ can catalyze the production of hydroxyl radicals (•OH) and superoxide anions (•O_2_^–^), destroying the phospholipid bilayer and proteins on the bacterial surface. This catalytic effect was significantly enhanced with light irradiation. We further tested the antibacterial efficacy of d-BN-Cu under sunlight by using the colony-forming unit (CFU) counting method with a solar simulator. The results showed that d-BN-Cu_1_, with just 0.26 at. % Cu content, could inactivate *E. coli* at 10^6^ CFU mL^–1^ within 15 min. This synergistic antibacterial platform was also effective against Gram-positive bacteria, highlighting its broad-spectrum potential. The findings in this work suggest that d-BN-Cu could serve as a promising framework for the prevention, control, and treatment of drug-resistant bacterial infections. Finally, for comparison, we studied other common transition metal elements in BN besides Cu: Cr, Mn, Fe, Co, Ni, and Zn, which cover most first-row transition metals.

## Results and Discussion

### Synthesis and Characterization

BN was pretreated by cryomilling for 120 min to produce defective BN (d-BN). Cryomilling takes advantage of conventional milling and a cryogenic N_2_ environment so that the milling time can be significantly reduced and oxidation can be substantially suppressed.^33^ This process introduced more defects and anionic functional groups, enhancing its reducibility, as seen in Figure 1a. To prepare Cu-loaded d-BN, 500 mg of d-BN was added to 10 mL of CuSO_4_ solution at varying concentrations (0.01, 0.005, 0.001, and 0.0005 M) and shaken for 3 h to ensure thorough mixing. The mixture was then centrifuged at 8000 rpm, the supernatant discarded, and the pellet rinsed with 10 mL of deionized water. The washing and centrifugation process was repeated three times, and the sediment was retained after the third centrifugation. The resulting samples were dried in a constant-temperature oven at 60 °C for 2 days. The d-BN samples were labeled d-BN-Cu_1_ to d-BN-Cu_4_, in descending order, based on the CuSO_4_ solution concentration. Similar to the above method, d-BN samples were loaded with other metals using salt solutions at 0.01 M; the salt solutions included CrCl_3_, MnCl_2_, FeCl_3_, CoCl_2_, NiCl_2_, and ZnCl_2_.

**Figure 1:**
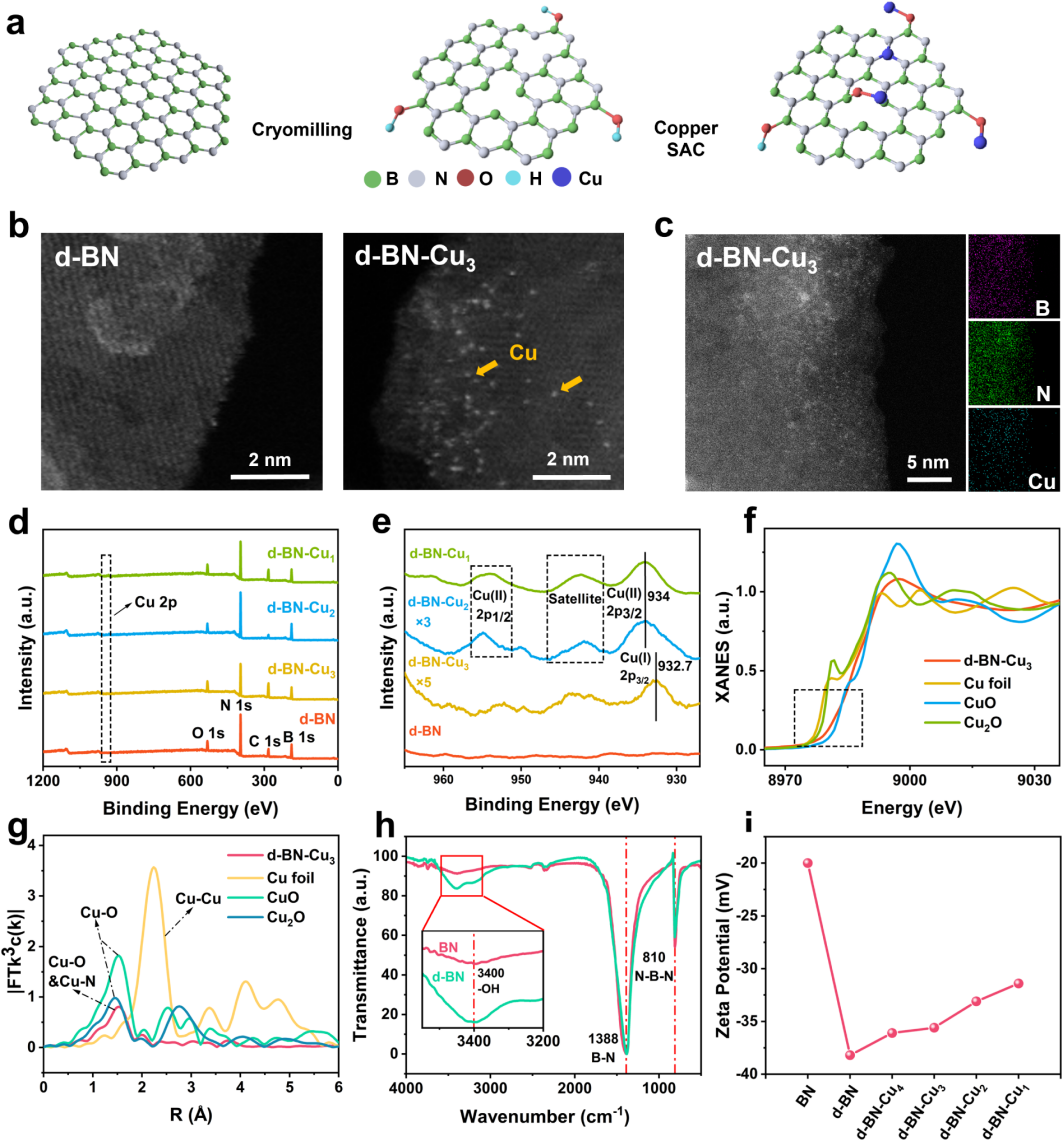
(a) Schematic illustration of the synthesis method of d-BN-Cu, (b) HAADF-STEM images of d-BN and d-BN-Cu_3_, and (c) EDS mapping showing the uniform distribution of Cu on d-BN-Cu_3_ nanosheets. (d) XPS spectra of d-BN, d-BN-Cu_1_, d-BN-Cu_2_, and d-BN-Cu_3_. (e) XPS spectra of Cu 2p of d-BN, d-BN-Cu_1_, d-BN-Cu_2_, and d-BN-Cu_3_ (due to the low Cu content, we smoothed the signal and increased the signal intensityof d-BN-Cu_2_ by three times, and increased the signal intensity of d-BN-Cu_3_ by five times). (f) The normalized Cu K-edge XANES spectra of d-BN-Cu_3_, (g) corresponding FT-EXAFS fitting curves of d-BN-Cu_3_. (h) FTIR of BN and d-BN. (i) zeta potential of BN loaded with different Cu concentrations.

As shown in Table S1, the Cu content in d-BN-Cu_1_ through d-BN-Cu_4_ was determined by using inductively coupled plasma (ICP) analysis. Scanning electron microscopy (SEM) images in Figure S1 reveal that in BN samples without cryomilling, large BN flakes (≥400 nm) are prevalent. However, in d-BN and d-BN-Cu_3_ samples (which underwent cryomilling), these large flakes are rarely observed, with most particles measuring around 100–300 nm. This size reduction is attributed to the mechanical forces of cryomilling, which cause larger BN flakes to break apart more easily. The atomic force microscopy (AFM) images shown in Figure S2 further corroborate these findings, showing significant interlayer stacking in BN and d-BN. Occasionally, large BN nanosheets appear in the BN samples, with sizes consistently between 100 and 300 nm, consistent with SEM observations. By contrast, in the AFM images, d-BN-Cu_3_ appears more evenly dispersed and with minimal interlayer stacking, likely due to the spatial forces exerted by the Cu atoms on the surface, which inhibit stacking in solution. Spherical aberration-corrected high-angle annular dark-field scanning transmission electron microscopy (HAADF-STEM) images (Figure 1b) show a clean and clearly layered d-BN surface. After loading with Cu atoms, distinct isolated bright spots appear on d-BN-Cu_3_ (Cu, 0.024 at. %), corresponding to Cu SACs. These images indicate that the Cu atoms are attached to the BN nanosheets as single atoms with a tendency to accumulate near the edges of the layers (Figure S3a), possibly due to a higher concentration of defects in these regions. Energy dispersive spectroscopy (EDS) elemental mapping (Figure 1c) confirms the presence of Cu and its uniform distribution across the BN nanosheets. However, for sample d-BN-Cu_1_ (with higher Cu concentration, 0.26 at. %) there are numerous Cu nanoclusters with a 2 nm diameter on the BN surface (Figure S3b).

X-ray photoelectron spectroscopy (XPS) was employed to investigate the surface properties and chemical structures of the samples, as shown in Figure 1d,e. The characteristic Cu peaks reveal that at low Cu concentrations, Cu predominantly exists as low-valence Cu^+^ (932.7 eV). When the Cu content is higher, it appears primarily as high-valence Cu^2+^ (934 eV). In d-BN-Cu_3_, which has a lower Cu content, the Cu atoms exist in a mixed valence state with both Cu^+^ and Cu^2+^ forms, though Cu^+^ is predominant. In contrast, in d-BN-Cu_2_ and d-BN-Cu_1_, where the Cu content is higher, Cu^2+^ dominates. This indicates that d-BN preferentially reduces divalent Cu ions, but when the Cu content exceeds the reduction capacity of d-BN, excess Cu is loaded onto its surface in its original valence state. The XPS peaks for B, N, and O show minimal variation across the samples (Figure S4). Additionally, the elemental composition of the samples was analyzed via XPS (Table S2). Since XPS provides surface-level information, the measured Cu content is slightly higher than expected.

Based on the detection results of HAADF-STEM images (Figures 1b and S3a), we conclude that sample d-BN-Cu_3_ is highly likely to be loaded onto d-BN in a single-atom form. To further verify our hypothesis and analyze the valence bond and coordination information on d-BN-Cu_3_, X-ray absorption near-edge structure (XANES) and EXAFS measurements were performed, using metallic Cu, Cu_2_O, and CuO as references. As shown in the Cu K-edge absorption spectrum (Figure 1f), the rising edge position of d-BN-Cu_3_ lies between those of Cu_2_O and CuO, indicating that the Cu in d-BN-Cu_3_ exists in a mixed valence state between +1 and +2.^34,35^ This unsaturated coordination structure of metal atoms is known to enhance catalytic activity.^36^ In Figure 1g, the Fourier transform EXAFS (FT-EXAFS) spectrum provides insights into the coordination environment of Cu in d-BN-Cu_3_. The absence of a Cu–Cu scattering path (around 2.24 Å) confirms that Cu does not form Cu–Cu metallic bonds in d-BN-Cu_3_. Additionally, the lack of any significant peaks beyond the first near-neighbor main peak suggests that the Cu atoms are not crystallized, further supporting the conclusion that Cu is dispersed as SACs. Based on the information on bond length and valence state (XPS), it can be concluded that after reduction by d-BN, the presence of the Cu element is mainly dominated by monovalent Cu–O bonds (B–O–Cu in d-BN-Cu_3_), while the possibility of a small amount of Cu–N bonds cannot be ruled out.^35,36^

To further confirm the bonding of Cu atoms on the BN surface, we analyzed functional groups and surface potentials using Fourier transform infrared (FTIR) spectroscopy (Figure 1h)^33,37^ and zeta potential measurements, respectively. A significant enhancement in the hydroxyl group (−OH) signal at 3400 cm^–1^ was observed with increasing cryomilling time (Figure 1h). The transmittance at 3400 cm^–1^ across samples with different cryomilling durations was statistically analyzed (Figure 1h). The results indicate that as ball milling time increases, the −OH content does not increase indefinitely but rather fluctuates within a certain range (Figure S5). Concurrently, a decrease in the zeta potential (Figure 1i) indicates an increase in surface anionic functional groups. This indicates that cryomilling promotes the formation of more B–OH groups at the edges of d-BN, which are negatively charged in solution and facilitate the adsorption of positively charged Cu ions. With the continuous increase of Cu element, the increase in zeta potential also confirms the consumption of B–OH anionic functional groups by Cu. These pieces of information further confirm that B–O–Cu is the main atomic configuration of Cu in d-BN-Cu_3_. The distribution of Cu atoms observed under HAADF-STEM further supports this finding (Figure S3a). Consequently, most of the Cu ions in d-BN-Cu_3_ will combine with B−OH-rich areas to form B−O−Cu defect structures, and a small amount of Cu ions are reduced to form Cu−N at B vacancies.

### Antibacterial Activity

To assess the impact of metal atom loading on the antibacterial properties of BN, we evaluated the antibacterial rates against*E. coli*­(ATCC 25922) and*S. aureus*­(ATCC 6538) using the CFU counting method. As shown in Figure 2a, seven different transition metals (Co, Cr, Fe, Mn, Ni, Zn, and Cu) were loaded onto d-BN, but only Cu exhibited rapid and near-complete bacterial inactivation, underscoring its superior catalytic properties on BN nanosheets. The results for different transition metals were confirmed via DFT calculations (Figure S6). Refer to the computational results below for a detailed analysis of the Cu defect structures. After 3 h of cocultivation, the samples were inoculated onto Luria–Bertani (LB) medium. We then used the same method to test the antibacterial rates of d-BN with varying Cu loading amounts against*E. coli*­(Figure 2b) and*S. aureus*­(Figures 2c and S7). The results showed that the d-BN-Cu series samples had significant antibacterial effects on both Gram-negative and Gram-positive bacteria, and their antibacterial effects were positively correlated with the Cu loading concentration. Meanwhile, the inhibitory effect of the sample on *E. coli* was slightly stronger than that on*S. aureus*, which is likely due to the thicker cell wall and richer peptidoglycan layer of *S. aureus*, typical of Gram-positive bacteria, providing better protection compared to Gram-negative bacteria. To further investigate, we centrifuged the mixture of the d-BN-Cu_3_ group in Figure 2b, observed atomic-level changes on the sample surface using STEM, and confirmed the absence of Cu residues in the supernatant using ICP. EDS mapping and HAADF-STEM images (Figure S8) revealed that Cu was still present in the sample and maintained its monatomic form. Additionally, in the ICP test results (Table S3), there was no detectable signal of Cu. The presence of potassium ions in the solution was attributed to the lysis of bacterial cell membranes, providing further evidence of bacterial inactivation.

**Figure 2:**
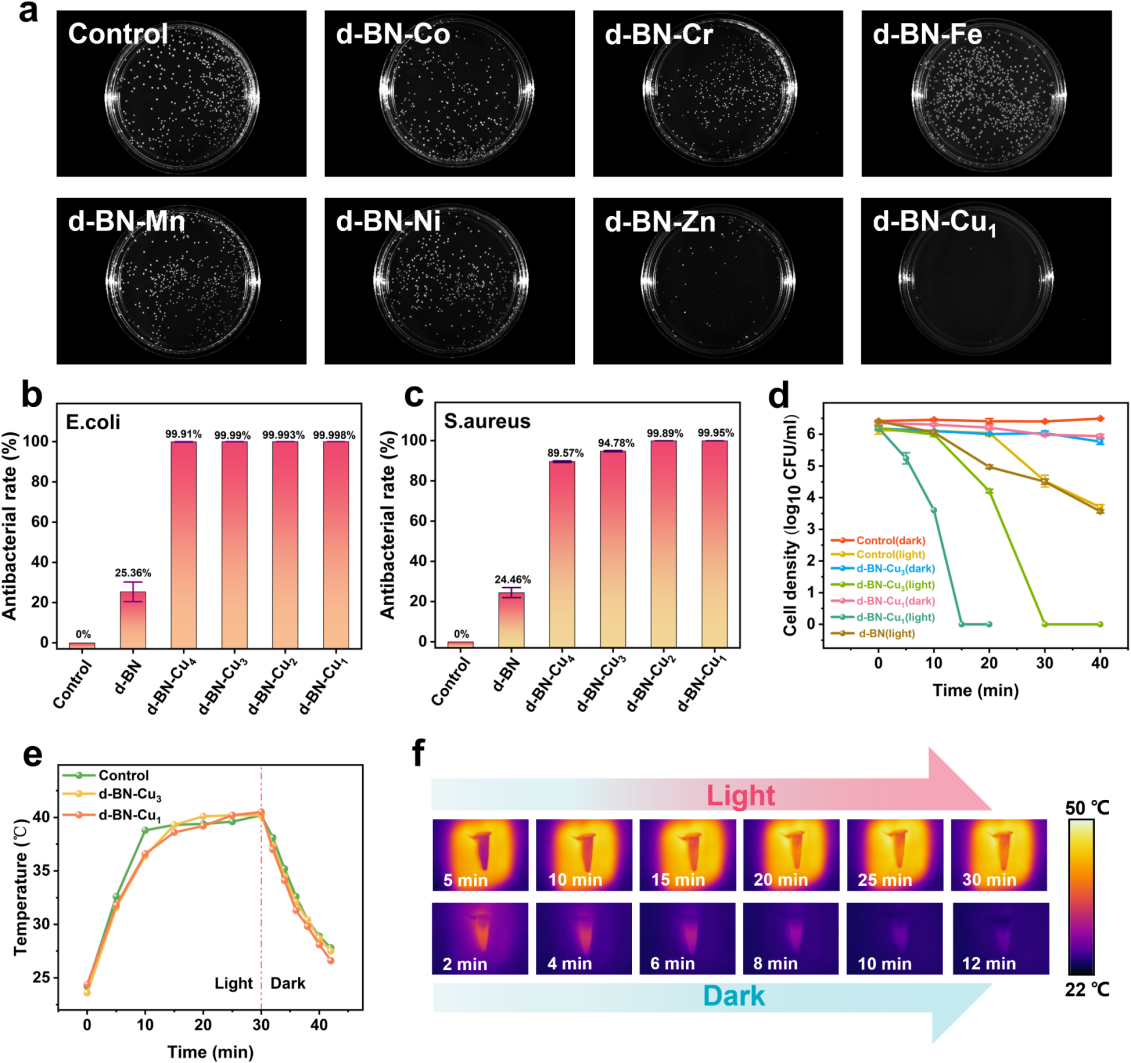
(a) Antibacterial activity of d-BN loaded with various metal ions (Co, Cr, Fe, Mn, Ni, Zn, Cu) against*E. coli*, assessed via colony counting. Cu-loaded d-BN (d-BN-Cu) shows the strongest bactericidal effect. The concentration of the bacterial solution was 10^6^ CFU mL^–1^, and it was incubated in a constant temperature shaker (37 °C/120 rpm) for 3 h. The mixed solution after incubation was diluted 100 times and inoculated for culture and counting. The antibacterial rate of d-BN, d-BN-Cu_1_, d-BN-Cu_2_, d-BN-Cu_3_, and d-BN-Cu_4_ against*E. coli*­(b) and*S. aureus*­(c). (d) Test of the antibacterial effect of d-BN, d-BN-Cu_3_, and d-BN-Cu_1_ on*E. coli*under simulated lighting conditions (400–1076 nm, AM 1.5 G, 100 mW cm^–2^). The dark group was treated at room temperature and kept away from light. The controls for the above inhibition experiments were a mixture of saline and bacterial solution, and the working concentration of the antibacterial material is 100 ppm. (e) Photothermal effects of the control, d-BN-Cu_1_, and d-BN-Cu_3_ at a concentration of 100 ppm under a solar simulator. (f) Photothermograms of d-BN-Cu_3_.

### Photocatalytic Disinfection Performance

We further investigated the antibacterial activity of samples against*E. coli*under sunlight using a solar simulator (Figure 2d). In the absence of light, d-BN-Cu_3_ exhibits no significant antibacterial effect; however, under illuminated conditions, it can completely inactivate 10^6^ CFU mL^–1^ of*E. coli*within 30 min. Meanwhile, the higher Cu content in d-BN-Cu_1_ enables bacterial elimination within 15 min, highlighting the beneficial role of Cu in the photocatalytic process and showcasing its potential for rapid, broad-spectrum antibacterial applications. Interestingly, we observed that even physiological saline, without the addition of antibacterial materials, demonstrated some antibacterial activity after 20 min of light exposure. To understand the system’s energy dynamics under illumination, we monitored temperature changes *in situ* using an infrared camera (Figure 2e, f). The temperature of the system quickly rises to 39 °C within the first 15 min of light exposure, after which the temperature increase slows down, reaching a maximum of approximately 41 °C. The increase in temperature leads to an accelerated microbial respiration rate,^38^ producing reactive oxygen species (ROS) that are harmful to cells.^39–41^ Consequently, even the control group, devoid of antibacterial materials, exhibits some bactericidal effects after prolonged light exposure. At the same time, there was not much difference in temperature between the groups, which also ruled out the influence of excessive temperature differences on the experimental control.

### Mechanistic Analysis

As illustrated in Figure 3a, the sterilization mechanism of d-BN-Cu can be categorized into two primary processes: (i) physical disruption of the cell membrane due to the sharp, atomic edges of the 2D material; and (ii) the generation of ROS by d-BN through the photocatalytic reaction of Cu, which disrupts the surface proteins and phospholipid bilayer of the cell. These actions, comprising physical cleavage and inactivation of active substances on the bacterial surface, interfere with the metabolic and proliferative activities of the cells, thus exerting a disinfecting effect. To analyze the mechanism of bacterial damage, we first observed the morphology of *E. coli* before and after treatment with d-BN-Cu_3_ using SEM (Figure 3b). The SEM images reveal membrane disruption after treatment with d-BN-Cu_3_. The extent of damage to the bacterial cell membrane often correlates with the size of the 2D material. The cryomilling process used in our work reduced the BN thickness to 6 nm (Figure S2), enhancing its ability to physically disrupt the bacterial cell membrane (Figures S9 and S10).

**Figure 3:**
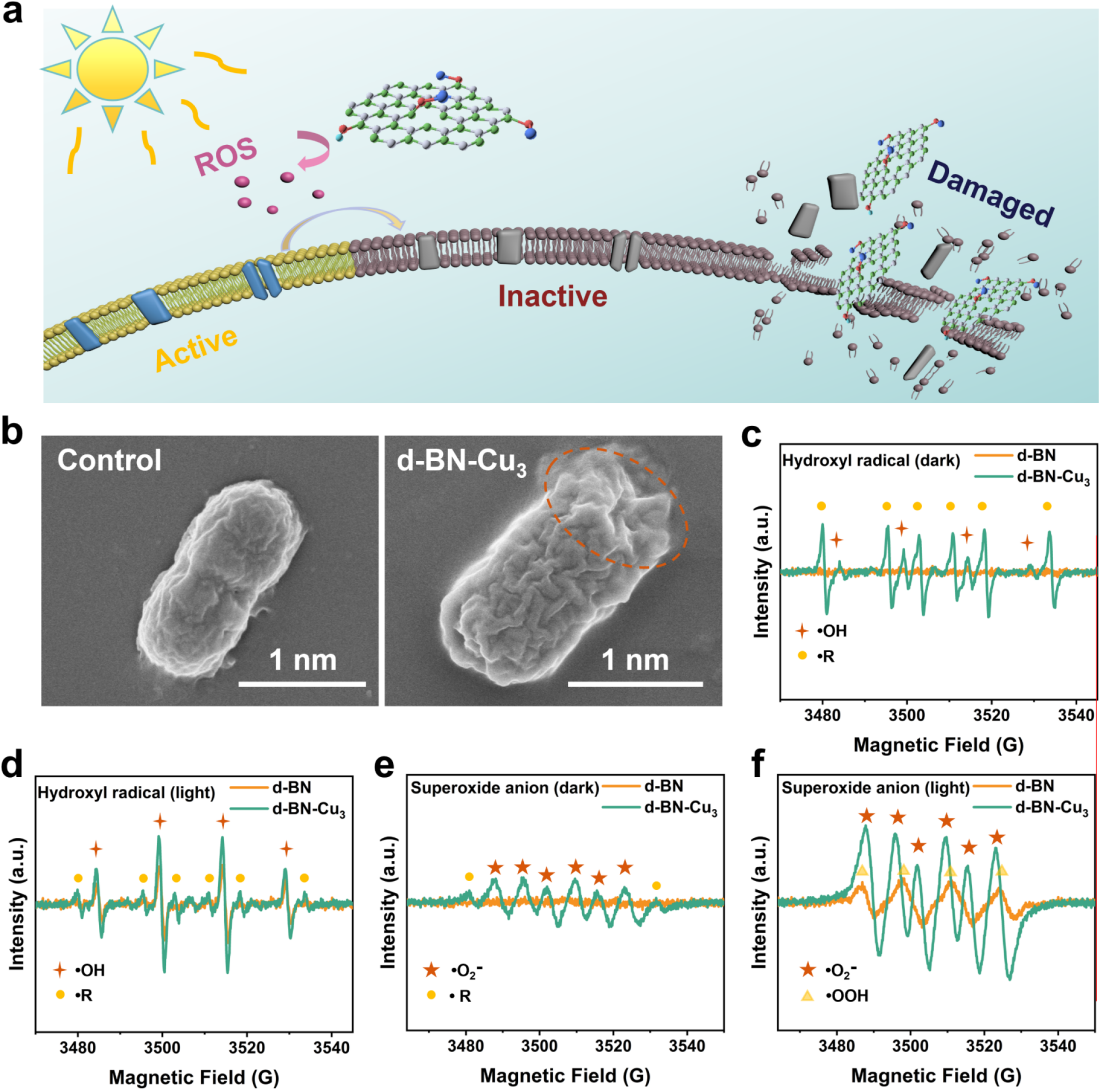
(a) Schematic diagram of the antibacterial mechanism of d-BN-Cu. (b) Morphology of *E. coli* before and after treatment with antibacterial materials under SEM. (c,d) EPR analysis of ROS generation by d-BN-Cu_3_ under illuminated and nonilluminated conditions, showing enhanced •OH production under light exposure. (e,f) Using EPR to test the catalytic effect of d-BN-Cu_3_ on •O_2_^–^ under light and no-light conditions.

The ROS formation catalyzed by the samples was evaluated using EPR, with the solvent alone serving as a blank control. In the absence of light (Figure 3c), no •OH peaks were detected in d-BN. However, a •OH peak was present in d-BN-Cu_3_, although not very strong. Interestingly, the d-BN-Cu_3_ sample also exhibited a strong peak of alkyl radicals (•R).^42^ This is because the capturing agent 5,5-Dimethyl-1-pyrroline N-oxide (DMPO) degrades under the influence of Cu^2+^ after capturing •OH, and the amount of •R generated is correlated with the pH.^43^ In illuminated conditions, the •OH peak intensity in d-BN-Cu_3_ significantly increased, while the peaks of •R notably decreased. It is worth noting that under illumination conditions, the •OH signal was also observed in the control group with only deionized water, and the signal was significantly enhanced in the presence of d-BN (Figure S11a). This indicates that under light conditions, d-BN has a catalytic effect on •OH. The intensity comparison of •OH is summarized in Table S4, and the peak intensity of the control group under light conditions is defined as 100% for quantitative comparison. In addition, the effects of d-BN loaded with six other transition group metals (Co, Cr, Fe, Mn, Ni, and Zn) on hydroxyl radical catalysis were evaluated and compared with d-BN-Cu_1_ (Figure S12). The EPR signal indicates that in the absence of light, aside from d-BN-Cu_1_, only d-BN-Mn exhibits a partial DMPO oxidation peak (DMPOO)^42^ and a special reactive oxygen peak (•SRO). The *G* value of •SRO is consistent with that of hydroxyl radicals (*G* ≈ 15); however, hydroxyl radicals have four peaks (peak ratio of 1:2:2:1), and •SRO has six peaks (peak ratio of 1:1:1:1:1:1). This could be due to unique metal-ion-DMPO interactions or a different ROS pathway, and it merits future investigation. After light exposure, it can be observed that the peak intensity of d-BN-Mn does not increase, indicating that it cannot continuously and stably produce •SRO. The catalytic effect of samples loaded with other transition group metals on hydroxyl radicals did not significantly improve under light irradiation; only the signal of d-BN-Zn was slightly enhanced under light irradiation (Table S5), indicating that Cu-loaded d-BN has the best catalytic effect on ROS.

The EPR signal of •O_2_^–^ is shown in Figure 3e,f. Without light, only d-BN-Cu_3_ exhibited a typical •O_2_^–^ peak along with weak •R peaks. After illumination, the signal intensity of d-BN-Cu_3_ markedly increased. Concurrently, the control and d-BN produced four peaks at similar positions (Figure S11b) and with similar intensities. This may be due to the overlap of •OOH peaks generated by the system under illumination. Previous EPR tests on the H_2_O_2_/UV system in methanol solvent exhibited peak positions similar to those in this experiment.^42^ Notably, we did not detect any singlet oxygen signals, even under light conditions (Figure S13). In summary, under light conditions, a certain amount of ROS will be produced in water, and d-BN can play a catalytic role in the production of •OH. Furthermore, BN doped with Cu catalyzes the generation of •OH and •O_2_^–^ more efficiently, achieving significant bactericidal effects and enhancing the catalytic reaction under light conditions.

### Biosafety Testing

Cell culture and cytotoxicity assays were used to evaluate the biocompatibility of d-BN-Cu (Figure S14), including MCF10A (normal human epithelial breast cell line), BEAS-2B (normal human bronchial epithelial cell line), and HPDE6-C7 (normal human pancreatic ductal epithelial cells). The IC_50_ values of d-BN-Cu for all three types of cells are higher than 100 ppm, indicating minimal toxicity overall. Notably, an increase in Cu loading is associated with increased toxicity. Based on the atomic distribution of Cu atoms, stability testing of Cu atom loading, and human cell toxicity testing, it has been shown that higher concentrations of Cu pose a greater risk of leakage and elevated biological toxicity, whereas single-atom Cu effectively mitigates these concerns. In the samples tested, the Cu atoms in d-BN-Cu_3_ exist in a single-atom form, exhibiting greater stability and reduced toxicity. Furthermore, they preserve excellent antibacterial properties. Consequently, further *in vivo* experiments are mainly focused on evaluating the single-atom configuration.

The effect of d-BN-Cu samples on inflammation rehabilitation *in vivo* was evaluated by using a mouse wound infection model (Figure 4). Ten μL of*E. coli*solution or*S. aureus*solution (10^6^ CFU mL^–1^) were dripped into mouse wounds to establish an infected wound model, and the mice were randomly divided into four groups to receive diverse treatments, including the control group, d-BN, d-BN-Cu_3_, and d-BN-Cu_1_. Figure 4a,b shows daily changes in the wound area of mice and reflects the impact of the sample on inflammation recovery. In the*E. coli*infection experiment (Figure 4a), due to the mild inflammation caused, the mice’s own immune system can quickly recover. Nevertheless, the wound recovery rate after treatment with d-BN-Cu_3_ and d-BN-Cu_1_ was still significantly faster than that of other groups. In the*S. aureus*infection experiment (Figure 4b,c), mice were more severely infected and required a longer recovery time. After 4 days of infection, it was found that the wound recovery rate of the mice treated with our sample was significantly better than that of the control group, and this recovery rate was positively correlated with the Cu content. Moreover, the healed skin was collected for histological analysis via hematoxylin and eosin (H&E) staining to evaluate the inflammation conditions (Figure 4d). In the control group, extensive necrosis was observed in the skin tissue, with a large number of inflammatory cells, such as granulocytes and lymphocytes, present. However, the number of inflammatory cells in the skin tissue of other experimental groups was relatively small, and the overall recovery level was higher than that of the control group. In summary, the d-BN-Cu_3_ sample we developed has good biocompatibility, minimal harm to human cells, and a positive effect on the recovery of bacterial infections and inflammation in the body.

**Figure 4:**
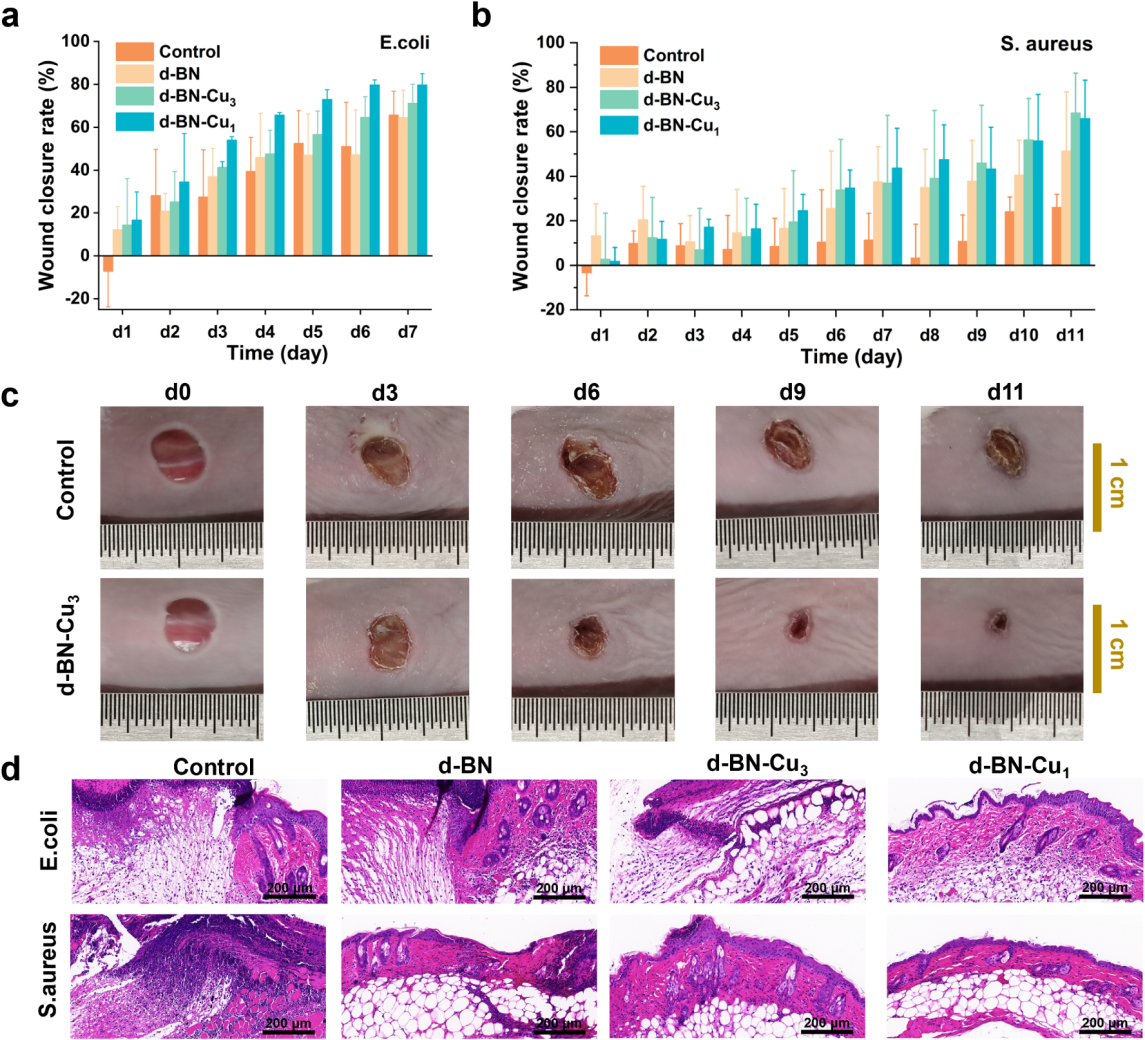
Statistical chart of the recovery rate of inflammatory wounds in mice infected with bacteria, including (a)*E. coli*and (b)*S. aureus*. On day 1 after the mice were infected with bacteria, they were grouped and inoculated with d-BN, d-BN-Cu_3_, d-BN-Cu_1_, and physiological saline (control). (c) The wound images of mice in the control group and d-BN group during the*S. aureus*infection experiment. (d) On day 3 postinfection, one mouse slice was taken from each group and stained with H&E.

### Theoretical Calculations

DFT calculations were performed to investigate the electronic properties of Cu SACs on BN. The optimized Cu coordination environment (B–O–Cu) enhances electron transfer and lowers the energy gap for ROS generation. Band structure analysis confirms that d-BN-Cu exhibits a reduced band gap (∼2.9 eV), facilitating visible-light-driven photocatalysis. Defect engineering simulations further revealed that BN vacancies contribute to Cu stabilization while modulating the electronic states for optimal redox reactions. These insights offer a design framework for next-generation antimicrobial SACs.

DFT results are plotted in Figure 5, showing the progression of the structural and electronic properties upon the addition of defects to the lattice. Figure 5a,c corresponds to the optimized geometries of different defect structures in a BN supercell. As defects are introduced, buckling occurs due to charge compensation and steric repulsions; this effect is more prevalent in the near vicinity of the defects. The defects in Figure 5a are calculated in a 4 **×** 4 supercell, and the structures in Figure 5c are calculated in a 5 **×** 5 supercell to avoid interactions between defective sites.

**Figure 5:**
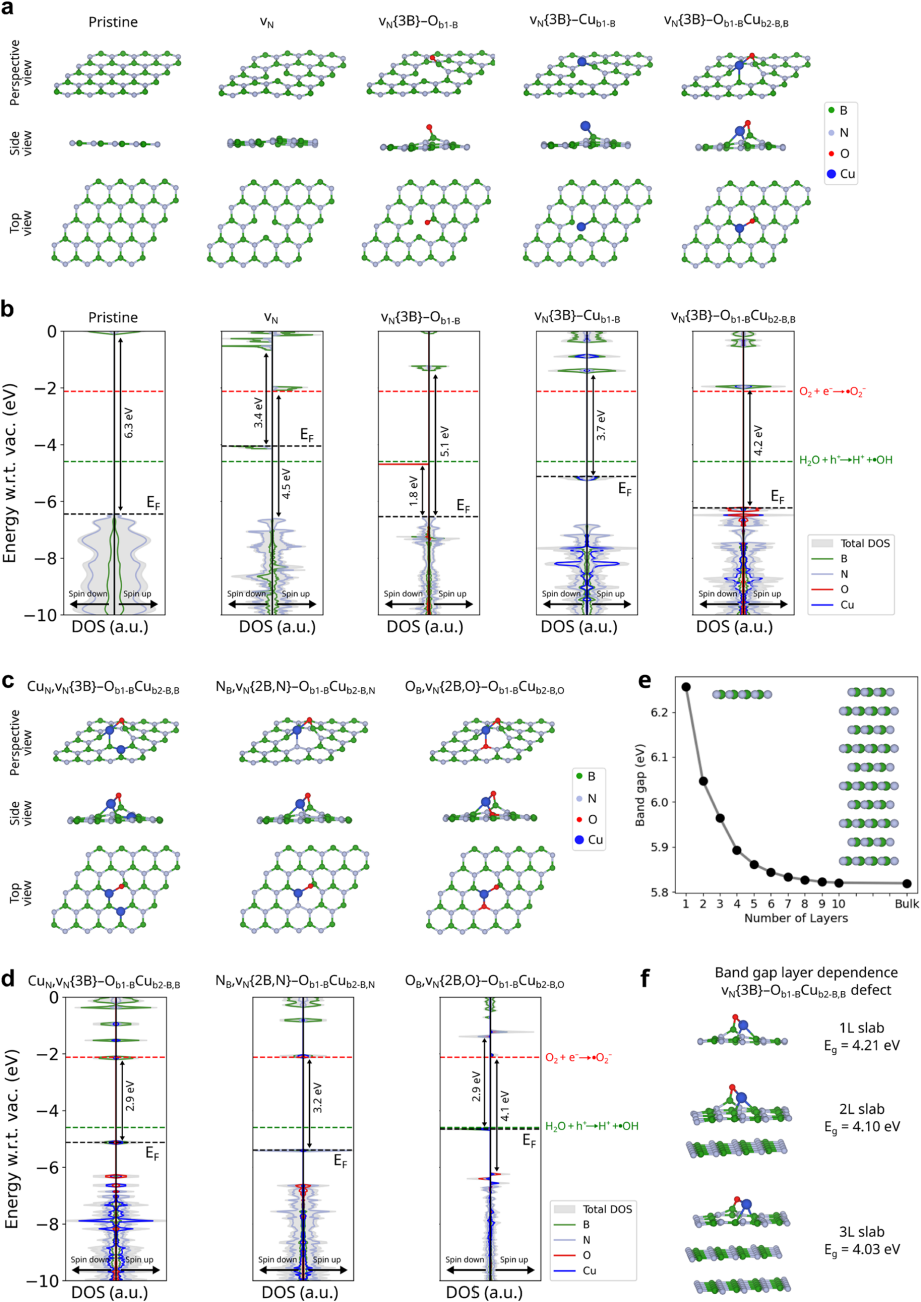
Progression of the structural and electronic features of BN as defects of interest are added to a supercell, calculated using hybrid density functional theory. (a) Optimized geometries (perspective, side, and top views) and (b) spin-polarized atom-projected density of states (DOS) aligned with respect to vacuum for the following structures: pristine, nitrogen vacancy (v_N_), nitrogen vacancy with oxygen attached to boron (v_N_{3B}–O_b1‑B_), nitrogen vacancy with Cu bonded to boron (v_N_{3B}–Cu_b1‑B_), and nitrogen vacancy with oxygen and Cu bonded to boron (v_N_{3B}–O_b1‑B_–Cu_b2‑B,B_). These defects are calculated in a 4 × 4 lattice. (c) Optimized geometries and (d) spin-polarized atom-projected DOS for “multidefects” of interest, taking the v_N_{3B}–O_b1‑B_–Cu_b2‑B,B_ defect as a starting point and adding Cu_N_, N_B_, and O_B_ to the lattice. These are hosted in 5 × 5 supercells. (e) Effect of layer number on the band gap for the pristine lattice (monolayer to bulk transition). (f) Effect of layer number on the v_N_{3B}–O_b1‑B_–Cu_b2‑B,B_ defect (one to three layers).

To study the structural effect of these defects, bond distances of interest for the following structures were measured and are shown in Table 1: nitrogen vacancy with O bonded to B (v_N_{3B}–O_b1‑B_); nitrogen vacancy with Cu bonded to B (v_N_{3B}–Cu_b1‑B_); nitrogen vacancy with O and Cu bonded to B (v_N_{3B}–O_b1‑B_–Cu_b2‑B,B_); defect including Cu substitution at an N site and nitrogen vacancy with O and Cu bonded to B (Cu_N_,v_N_{3B}–O_b1‑B_–Cu_b2‑B,B_); defect including N substitution at a B site and nitrogen vacancy with O and Cu bonded to B (N_B_,v_N_{2B,N}–O_b1‑B_–Cu_b2‑B,B_); and defect including O substitution at a B site and nitrogen vacancy with O and Cu bonded to B (O_B_,v_N_{2B,O}–O_b1‑B_–Cu_b2‑B,B_). The defect notation used in this paper is described in the Methods section.

**Table 1:** Bond Distances of Interest in the v_N_{3B}–O_b1‑B_, v_N_{3B}–Cu_b1‑B_, v_N_{3B}–O_b1‑B_–Cu_b2‑B,B_, Cu_N_,v_N_{3B}–O_b1‑B_–Cu_b2‑B,B_, N_B_,v_N_{2B,N}–O_b1‑B_–Cu_b2‑B,B_, and the O_B_,v_N_{2B,O}–O_b1‑B_–Cu_b2‑B,B_ Defect Structures^a^

Defect structure	B–O distance (Å)	B–Cu distance (Å)	O–Cu distance (Å)
v_N_{3B}–O_b1‑B_	1.37	-	-
v_N_{3B}–Cu_b1‑B_	-	1.98	-
v_N_{3B}–O_b1‑B_–Cu_b2‑B,B_	1.34	1.97	1.84
Cu_N_,v_N_{3B}–O_b1‑B_–Cu_b2‑B,B_	1.34	1.94	1.83
N_B_,v_N_{2B,N}–O_b1‑B_–Cu_b2‑B,N_	1.34	1.94	1.83
O_B_,v_N_{2B,O}–O_b1‑B_–Cu_b2‑B,O_	1.34	1.94	1.83, 1.94

a Structures were fully optimized in terms of atomic positions and lattice parameters.

From the values in Table 1, the B–O and B–Cu distances are smaller in the v_N_{3B}–O_b1‑B_–Cu_b2‑B,B_ defect compared to the individual v_N_{3B}–O_b1‑B_ and v_N_{3B}–Cu_b1‑B_ structures, indicating stronger defect interactions with the lattice in the v_N_{3B}–O_b1‑B_–Cu_b2‑B,B_ structure. This result predicts that the simultaneous addition of O and Cu to the structure (B–O–Cu coordination) has a synergistic effect, with more stable bonds than those of O or Cu by themselves. This is verified in Figure S15, which confirms that B–O–Cu defects are more stable than adsorbed Cu–Cu clusters by 0.012 eV/atom. Taking the structure with B–O–Cu coordination as a base, the addition of more defects to the lattice (Cu_N_, N_B_, and O_B_) results in even smaller B–Cu and O–Cu bond distances, showing a similar synergistic effect. Based on the chemical intuition gained from experiments and the exhaustive approach taken to calculate these multidefect structures (see Methods), we believe that these models present high stability and correctly capture the complexity found in our experimental samples.

Figure 5b,d shows the density of states (DOS) aligned with respect to vacuum (*w.r.t. vac.*) for the pristine and defective structures. This alignment serves as a guide to analyze how suitable these structures are for performing reactions of interest. The red and green lines in the DOS plots correspond to the redox potentials for O_2_ reduction (−2.12 eV *w.r.t. vac.*) and H_2_O oxidation (−4.60 eV *w.r.t. vac.*) for the production of •O^2–^ and •OH, respectively. In general, materials with a conduction band minimum (CBM) above the O_2_ reduction potential and a valence band maximum (VBM) below the H_2_O oxidation potential can generate electrons/holes with favorable energies to catalyze these reactions. Furthermore, their band gap determines the photon energy required to excite an electron from the valence to the conduction band and also serves as a design principle. Based on our experimental design for the antibacterial tests (see Methods), photon wavelengths fall between 400 and 1100 nm (3.1 to 1.13 eV). Thus, we are particularly interested in materials with band gaps ≤ 3.1 eV.

Starting our analysis with the pristine BN lattice, we observe that its band alignment is appropriate for carrying out both redox reactions. However, the pristine monolayer is an insulator with a band gap of ≈6.3 eV, impeding photoexcitation and discarding it for ROS production. Introducing nitrogen vacancies (**v**_**N**_) drastically changes the DOS profile, resulting in the appearance of intermediate states, which arise mainly from boron contributions. These intermediate states fall directly above the O_2_ reduction potential (red dashed line) in the spin-up channel and directly below the Fermi level in the spin-down channel. The DOS profile now has two spin-polarized band gaps of 4.5 eV (spin-up) and 3.4 eV (spin-down). The spin-up channel has an appropriate alignment for carrying out both ROS reactions (•O^2–^ and •OH production), although its band gap of 4.52 eV limits electronic excitation. The spin-down channel has a smaller band gap, but its alignment only allows for the production of •O^2–^, since its valence band is now mediated by the intermediate defect levels. Moreover, the large gaps below the valence band might prevent the conduction of electrons within the structure, slowing the catalytic process. We note that the concentration of vacancies in a 4 **×** 4 supercell is 3.22 at%. Nonetheless, similar results are found when using 5 **×** 5 and 6 **×** 6 supercells with vacancy concentrations of 2.04 and 1.41 at. % (see Figure S16).

The addition of oxygen to the vacancy structure (**v**_**N**_ **{3B}–O**_**b1‑B**_) again changes the DOS profile, where it now exhibits intermediate spin-polarized defect states from oxygen contributions in the spin-down channel. This structure has an appropriate band alignment for the production of ROS in the spin-up channel but presents a large band gap of ≈5.1 eV, hindering electronic photoexcitation. The spin-down channel has a smaller band gap of ≈1.8 eV but does not present appropriate band alignment for the generation of ROS, since the CBM now corresponds to the intermediate energy levels, which fall below the oxidation potential for •OH production. On the other hand, Mulliken Population Analysis reveals that the exposed oxygen has one unpaired electron, likely presenting high reactivity. Based on the experimental FT-EXAFS results—which show that the coordination of Cu in d-BN-Cu_3_ is with oxygen or nitrogen—we conclude that this exposed oxygen likely serves as a binding site for Cu, rather than as a catalytically active site. On the other hand, Cu addition to the vacancy structure (**v**_**N**_ **{3B}–Cu**_**b1‑B**_) has appropriate band alignments for both ROS, but the DOS shows a band gap of ≈3.7 eV, which falls outside of our range of interest, preventing the excitation of electrons and hindering its ability for photocatalysis.

The next defect of interest has both oxygen and Cu bonded to boron at the vacancy site (**v**_**N**_ **{3B}–O**_**b1‑B**_ **–Cu**_**b2‑B,B**_). This structure results in appropriate band alignment for both ROS (•OH and •O_2_^–^) and reduced gaps below the VBM, resulting in enhanced available electronic states within the material. These electronic properties predict that the v_N_{3B}–O_b1‑B_–Cu_b2‑B,B_ structure, with B–O–Cu coordination, presents enhanced catalytic activity for hydroxyl and superoxide ROS. Further, we calculated 5 **×** 5 (1.96 at. % Cu content) and 6 **×** 6 (1.36 at. % Cu content) supercells hosting the v_N_{3B}–O_b1‑B_–Cu_b2‑B,B_ defect, yielding similar results (see Figure S17). However, similar to the previous structure, its band gap of 4.2 eV prevents photoexcitation within our range of interest.

Taking the aforementioned structure with B–O–Cu coordination as a base, we generated multidefect structures of interest by adding Cu_N_, N_B_, and O_B_ to the lattice (Figure 5c). The DOS values for these structures are shown in Figure 5d. These defects were hosted in a 5 **×** 5 supercell, and they capture more of the complexity expected in cryomilled samples. The structures that incorporate Cu substituting nitrogen (**Cu**_**N**_,**v**_**N**_ **{3B}–O**_**b1‑B**_ **–Cu**_**b2‑B,B**_) and nitrogen substituting boron (**N**_**B**_,**v**_**N**_ **{2B,N}–O**_**b1‑B**_ **–Cu**_**b2‑B,N**_) have an equal distribution of spin-up and spin-down electrons. They both present correct band alignment for the production of ROS and reduced band gaps (2.9 and 3.2 eV for Cu_N_,v_N_{3B}–O_b1‑B_–Cu_b2‑B,B_ and N_B_,v_N_{2B,O}–O_b1‑B_–Cu_b2‑B,O_, respectively). The final defect of interest is **O**_**B**_,**v**_**N**_ **{2B,O}–O**_**b1‑B**_ **–Cu**_**b2‑B,O**_, which presents an uneven distribution of electrons along the spin-up and spin-down channels. The spin-up band gap value is outside of our range of interest at 4.1 eV; however, the spin-down band gap has both a correct band gap value (2.9 eV) and alignment to produce ROS. These three multidefect calculations show viable electronic structures for ROS formation in our energy range of interest. In summary, based on the comprehensive computational analysis of various defect structures, multidefect systems incorporating B–O–Cu coordination complexes in conjunction with additional Cu_N_, N_B_, and O_B_ lattice defects emerge as the most probable candidates for ROS generation. This conclusion is supported by the favorable alignment of redox potentials and band gap energies within the target range for photocatalytic ROS activity.

The calculations shown thus far explore defect formation in a single layer of BN. However, additional calculations were performed for multilayer structures to elucidate the effect of layer number on electronic properties. Figure 5e shows the monolayer-to-bulk transition for pristine BN, showing a band gap difference of 0.44 eV between the monolayer and bulk, with several intermediate values. A similar band gap reduction is observed in Figure 5f, which shows the effect of adding layers to the v_N_{3B}–O_b1‑B_–Cu_b2‑B,B_ defect, showing a reduction of ≈0.2 eV between monolayer and three-layer slabs. These results indicate that the band gap can be engineered through the layer number. Since cryomilling likely yields slabs of different thicknesses, we believe that this band gap modulation via layer addition/subtraction can also be an important contributor to the photocatalytic activity observed experimentally.

## Conclusion

In this study, we successfully developed Cu single-atom catalysts (d-BN-Cu_3_) with enhanced photocatalytic properties. Boron nitride (BN), known for its exceptional chemical stability, was employed as a substrate to anchor Cu atoms in a single-atom form, ensuring high catalytic efficiency while preventing Cu dissolution and reducing its biological toxicity. The d-BN-Cu system effectively catalyzes the production of reactive oxygen species (ROS), such as hydroxyl radicals (•OH) and superoxide anions (•O_2_^–^), which can damage bacterial cell membranes and genetic material, leading to rapid bacterial inactivation. Meanwhile, under illumination, the efficiency of the d-BN-Cu catalytic generation of ROS will be significantly improved. *In vivo* experiments have shown that d-BN-Cu can effectively promote the recovery of inflammation caused by bacteria. Density functional theory (DFT) simulations played a pivotal role in understanding the structural characteristics and catalytic mechanisms of d-BN-Cu. The simulations not only identified the optimal coordination environment (B–O–Cu with additional Cu_N_, N_B_, and O_B_ defects) for maximizing ROS catalytic activity but also provided insights into the atomic-level interactions between Cu atoms and the BN substrate. Computational modeling allowed us to identify structures that yield the target band gap and redox alignments for ROS generation, validating the experimental results by elucidating the electronic mechanisms and adsorption processes through which d-BN can provide antibacterial activity. Moreover, the DFT results guided the development of future design principles for ROS catalysis in single-atom catalysts (SACs), highlighting how the strategic manipulation of coordination structures can enhance catalytic performance. Under light irradiation, the photocatalytic efficiency of d-BN-Cu_3_ is significantly enhanced, achieving rapid inactivation of*E. coli*at a concentration of 10^6^ CFU mL^–1^ within just 30 min, ensuring a high antibacterial effect while also considering safety during use. Our findings underscore the importance of optimizing interactions between active catalytic sites and their substrates to fully exploit the catalytic potential of the material. The unique planar structure of two-dimensional materials, such as BN, presents vast opportunities for advancing SAC technologies. This study provides not only a deeper understanding of the structural and mechanistic aspects of ROS catalysis but also a promising pathway for the design of efficient and sustainable antibacterial systems. Furthermore, the insights gained from this work offer a new strategy for addressing the environmental challenges posed by drug-resistant bacteria.

## Methods

### Cryomilling

3.5 g boron nitride was cryomilled in a polycarbonate-encapsulated cell (SPEX 6775 Freezer/Mill) at −196 °C. The polycarbonate-encapsulated compactor (SPEX 6775) hits the two ends of the cell with a frequency of 10 Hz (cps). Four samples were prepared with different cryomilling times: 45, 90, 120, and 150 min.

### Metal Reduction

d-BN-Cu: 500 mg of d-BN was added to 10 mL of CuSO_4_ solution at varying concentrations (0.01, 0.005, 0.001, and 0.0005 M) and shaken for 3 h to ensure thorough mixing. The mixture was then centrifuged at 8000 rpm, the supernatant discarded, and the pellet rinsed with 10 mL of deionized water. The flushing and centrifugation process was repeated three times, and the sediment was retained after the third centrifugation. The resulting samples were dried in a constant-temperature oven at 60 °C for 2 days. The d-BN samples were labeled d-BN-Cu_1_ to d-BN-Cu_4_ in descending order based on the CuSO_4_ solution concentration. Similar to the above method, d-BN samples were loaded with other metals using salt solutions at 0.01 M. The salt solutions included CrCl_3_, MnCl_2_, FeCl_3_, CoCl_2_, NiCl_2_, and ZnCl_2_).

### Antibacterial Experiment

Cultivate*E. coli*in LB medium for 3–4 h to obtain a mixed bacterial solution of 10^9^ CFU mL^–1^. Afterward, centrifuge at 12000 rpm and wash with physiological saline to remove excess residue, repeating the above operation 3 times. Dilute the cleaned bacterial solution with physiological saline to OD600 ≈ 0.14, at which point the bacterial concentration is approximately 10^8^ CFU mL^–1^. Weigh and prepare 5 mL of 100 ppm d-BN-Cu_3_ solution and sonicate for 10 min. Then, 0.1 mL of the diluted bacterial solution was added to obtain a final bacterial concentration of approximately 10^6^ CFU mL^–1^. The control group added the bacterial solution directly to physiological saline without adding any materials. Incubate in a constant temperature shaker (120 rpm, 37 °C) for 3 h. Finally, the bacterial concentration of the samples was measured using standard spread plating techniques.

### Photocatalytic Antibacterial Experiment

The photocatalytic antibacterial experiment used a solar simulator to simulate sunlight exposure. A solar monocrystalline silicon solenoid valve was used to calibrate the light intensity to one solar light (Zolix, Sirius-SS300A-L, 400–1076 nm, AM 1.5 G, 100 mW cm^–2^). Then, the material was dispersed in 1 mL of bacterial solution, with the same concentration (100 ppm) of material and bacterial solution (10^6^ CFU mL^–1^) as in the antibacterial experiment described above. After a period of light exposure, immediately inoculate on LB medium, incubate, and count using the above method. The bacterial concentration of the sample was measured using standard coating techniques after experiencing specific lighting conditions. The infrared camera recorded the temperature changes of the system during the light antibacterial experiment. This experiment is an independent experiment, and the relevant parameters of the experiment are the same as those of the light antibacterial experiment. Temperature recording is divided into two stages: (i) temperature rise stage (recorded every 5 min), and (ii) temperature drop stage (recorded every 2 min).

### SEM Observation of Bacterial Morphology

Take 4 mL of*E. coli*suspension with OD600 ≈ 1 and add 1 mL of 500 ppm d-BN-Cu_3_ to it. Incubate in a constant temperature shaker (120 rpm, 37 °C) for 3 h. Take a 1 mL portion of the mixed solution, centrifuge it (12000 rpm), and and remove the supernatant. The cells were then fixed with 1 mL of 2.5% glutaraldehyde for 12 h. The cells were dehydrated with 30%, 50%, 70%, 90%, and 100% ethanol solutions. After that, it was diluted 10-fold with anhydrous ethanol and dripped onto the silicon wafers to dry naturally. A 1500 nm Au film was coated on the sample surface, and the bacterial morphology was observed under SEM.

### Detection of ROS

The signals of the three ROS and their changes under light conditions were detected using EPR. A 200–2000 nm xenon lamp was used as a light source during the detection. The samples to be tested were 500 ppm of 120 BN and d-BN-Cu_3_, respectively, and the control was a solvent without any added material. For the detection of •OH, 5,5-Dimethyl-1-pyrroline N-oxide (DMPO, 100 mM) was chosen as the trapping agent, and deionized water was chosen as the solvent. The signals were determined before and after 1 min of light exposure, respectively. For the determination of the superoxide anion, DMPO (100 mM) was also used as the capture agent, and methanol as the solvent. The signals were determined before and after 1 min of light exposure, respectively. For the determination of monoclinic states, 2,2,6,6-Tetramethyl-4-piperidone hydrochloride (TEMP, 20 mM) was selected as the capture agent, and deionized water was used as the solvent. The signals were determined before and after 1 min of light exposure, respectively. If there are parameter changes in individual experiments, additional explanations will be provided in the caption.

### Cell Culture and Cytotoxicity Assay

MCF10A (normal human epithelial breast cell line) was purchased from Pricella Biotechnology Co., Ltd. (Wuhan, China). HPDE6-C7 (normal human pancreatic ductal epithelial cells) was purchased from Jennio Biotech Co., Ltd. (Guangzhou, China). BEAS-2B (normal human bronchial epithelial cell line) was obtained from Peter Lobie’s lab. MCF10A cells were grown in DMEM/F12 supplemented with 5% horse serum, 20 ng/mL EGF, 0.5 μg/mL hydrocortisone, 10 pg/mL insulin, 1% NEAA, and 1% penicillin-streptomycin at 37 °C in a humidified incubator containing 5% CO_2_; BEAS-2B cells were grown in DMEM medium supplemented with 10% FBS and 1% penicillin-streptomycin at 37 °C in a humidified incubator containing 5% CO_2_; HPDE6-C7 cells were grown in MEM medium supplemented with 10% FBS and 1% penicillin-streptomycin at 37 °C in a humidified incubator containing 5% CO_2_. A cytotoxicity assay was performed on HPDE6-C7, MCF10A and BEAS-2B cells. In brief, 5 × 10^3^ cells were plated in a 96-well plate overnight, and the cell viability was measured according to the alamarBlue (Invitrogen, DAL1025) instructions after 72 h of exposure to diluted compounds (0, 0.01, 0.1, 1, 10, 100 μg/mL dissolved in saline) in the medium. The control groups were cultured in medium with equal volumes of saline, and no less than four replicate wells were set up for each group. Ten μL of alamarBlue was added to each well, and the fluorescence was measured using an excitation at 535 nm and an emission at 590 nm using a microplate reader (Tecan SPARK 10M, Switzerland). All experiments were performed in medium with 2% serum.

### In Vivo Experiments

Anesthetize mice with isoflurane gas, and shave the hair on the back area above the tail. After disinfecting the skin, except for the control group, all other groups of mice were cut into circular wounds with a diameter of 5 mm on their back skin. Take a suspension of*E. coli*or*S. aureus*­(10^7^ CFU mL^–1^, 10 μL) and drop it onto a mouse wound to simulate inflammation. After 24 h of bacterial infection in mice, a control experiment was conducted by adding antibacterial solution (100 ppm, 100 μL) or an equal amount of physiological saline to the wound site. At the same time, photographs were taken every day and the wound area of each group of mice. ImageJ image analysis software quantifies the wound area and calculates the wound healing rate. Healing rate = (Day 0 wound area – Day *n* wound area)/Day 0 wound area × 100%. On the second day after treatment, one specimen was taken from each group for separation of the back wound tissue and fixed with 4% paraformaldehyde solution for H&E staining.

### Defect Notation

The defect notation shown in this article is described by the following rules, which allow for an explicit and descriptive nomenclature.

**v**_**X**_: vacancy of atom X

{}: denotes exposed atoms with dangling bonds on the surface

**X–Y**: bonding between atoms X and Y

**b1-X** (used as subscript): monodentate binding with surface atom X

**b2-X,Y** (used as subscript): bidentate binding with surface atoms X and Y

For example, the **v**_**N**_ **{3B}–O**_**b1‑B**_ **–Cu**_**b2‑B,B**_ defect denotes a nitrogen vacancy, which results in three exposed boron atoms. Additionally, oxygen binds to one exposed boron atom in a monodentate fashion, Cu binds to this oxygen, and also has a bidentate bond with two surface boron atoms. Multiple defects are appended to the notation with a comma. For example, adding a nitrogen substitution at a boron site to the above example would yield: N_B_, **v**_**N**_ **{3B}–O**_**b1‑B**_ **–Cu**_**b2‑B,B**_.

### Theoretical Quantum Simulations

Calculations were carried out using unrestricted hybrid Density Functional Theory within the CRYSTAL23 code,^44^ which employs Gaussian-type orbitals (GTOs), allowing an efficient implementation of hybrid DFT. The exchange-correlation functional was defined using the formulation parameterized by Heyd, Scuseria, and Ernzerhof^45^ (HSE06), which was used for all geometry optimization and single-point energy calculations. Long-range interactions were taken into consideration via Grimme’s third-order (−D3) dispersion corrections.^46^ BSSE-corrected double-ζ with polarization (DZVP) quality basis sets were employed for structural optimizations, and triple-ζ with polarization (TZVP) quality basis sets were used to compute total energies and electronic properties.^47^ The boron nitride layers were separated by a distance of ≈ 500 Å (the default parameter for slabs in CRYSTAL23), resulting in virtually no periodicity in the *z*-direction. This separation is possible due to the use of localized Gaussian orbitals. Defect calculations were performed in 4 × 4 and 5 × 5 supercells (defect concentration of ≈2–4 at. %), ensuring approximately 10 Å between defects. Extra selected calculations, shown in the Supporting Information, were carried out in larger 6 × 6 supercells (defect concentration of ≈1–2 at. %). All structures were fully optimized in their atomic positions and lattice parameters. The thresholds used for evaluating the self-consistent field energy convergence, root mean squared (RMS) forces, maximum forces, root mean squared atomic displacements, maximum atomic displacements, and between-geometry convergence energies were set to 2.72 × 10^–6^ eV, 1.54 × 10^–2^ eV/Å, 2.31 × 10^–2^ eV/Å, 6.35 × 10^–4^ Å, 9.53 × 10^–4^ Å, and 2.72 × 10^–6^ eV, respectively. Electronic spin states were allowed to relax in the unrestricted wave function calculations. The k-points used to compute the Hamiltonian matrix were sampled using a Pack–Monkhorst grid with a resolution approximately between $\frac{1}{b_{i}}\frac{2\pi }{40}$ and $\frac{1}{b_{i}}\frac{2\pi }{60}$ , where *b*_*i*_ are reciprocal lattice vectors. On the other hand, the density matrix and Fermi energy were computed on a denser Gilat k-point grid, with a resolution between $\frac{1}{b_{i}}\frac{2\pi }{80}$ and $\frac{1}{b_{i}}\frac{2\pi }{120}$ . Band edges were aligned with respect to vacuum by shifting the Fermi level relative to the vacuum level, which is defined as the asymptotic value of the plane-averaged electrostatic potential sufficiently far away from the slab (approximately 50 Å). The description of the electrostatic potential was improved by adding ghost atoms in the near vicinity of the slabs’ outermost layers. We note that band edge positions may shift in an experimental setting, namely, due to different pH conditions, or charge accumulation, depletion, and inversion. In this regard, the electron and hole quasi-Fermi levels—which depend upon the concentration of defects, temperature, and sample size—might serve as more accurate descriptors of photocatalytic activity.^48^ However, the calculations shown in this work serve as a guide for understanding the underlying electronic structure that gives rise to the experimentally observed formation of ROS.

### Computational Defect Structure Generation

Defects have the potential to form in almost any lattice position. Therefore, to correctly describe the phenomena that occur in nature, defect generation must be carried out in a way that spans a majority of the defect space. To generate defective supercells computationally, we rely on chemical information obtained by experiments and leverage that information by taking an exhaustive approach that utilizes symmetry to explore defect space in a computationally efficient way. The methodology used in this work was developed by Davidsson et al. as a module in the Atomic Simulation Environment,^49^ called DefectBuilder.^50^ This tool allows quick generation of defective supercells, hosting defects at different positions in the structure and avoiding the repetition of symmetrically equivalent structures. This is particularly useful when dealing with double defects.

### Defect Stability Screening

After the structures were generated with DefectBuilder (on the order of hundreds for this work), single-point calculations were carried out. DFT energies for stoichiometric structures with defects incorporated in different sites were directly compared. The three most stable structures for each stoichiometry were selected and their geometries were fully optimized. After these optimizations, only the lowest-energy structure was selected and is shown in this work.

## Supplementary Material



## Notes

Information about the instruments used in the experiment, the reagents used in the experiment, determination of Cu content using ICP (**Table S1**), XPS analysis of material element content (**Table S2**), determination of Cu and K ions in the supernatant of the mixture after sterilization by BN using ICP (**Table S3**), statistical summary of EPR signals of hydroxyl radicals in Figure 3d (**Table S4**), statistical summary of EPR signals of hydroxyl radicals in **Figure S12** (**Table S5**), SEM images of BN, d-BN, and d-BN-Cu_3_ (**Figure S1**), AFM images of BN, d-BN, and d-BN-Cu_3_ (**Figure S2**), HAADF-STEM images of d-BN-Cu_3_ and d-BN-Cu_1_ (**Figure S3**), X-ray Photoelectron Spectroscopy (XPS) spectra of d-BN, d-BN-Cu_1_, d-BN-Cu_2,_ and d-BN-Cu_3_ (**Figure S4**), FTIR signals of BN with different cryomilling times were detected, and the transmittance at 3400 cm^–1^ was statistically analyzed (**Figure S5**), computational optimized geometries and DOS for defect structures with different transition metals (**Figure S6**), evaluation of the antibacterial performance of d-BN-Cu (100 ppm) against *S. aureus* using the plate colony counting method (**Figure S7**), HAADF-TEM image of d-BN-Cu_3_ (used) and EDS mapping of d-BN-Cu_3_ (used) (**Figure S8**), effect of different cryomilling times on the antibacterial rate of BN on *E. coli* and *S. aureus* (**Figure S9**), morphology of *E. coli* before and after treatment with d-BN and d-BN-Cu_3_ under SEM (**Figure S10**), EPR analysis of the catalytic effect of control and d-BN on •OH and •O_2_^–^ (**Figure S11**), the influence of d-BN loaded with seven different transition group metals (Cu, Mn, Fe, Co, Ni, Zn, Cr) on the EPR signal of hydroxyl radicals (**Figure S12**), EPR analysis of the catalytic effect of d-BN and d-BN-Cu_3_ on singlet oxygen under light and no-light conditions (**Figure S13**), cytotoxicity assay of d-BN-Cu for MCF10A, BEAS-2B, and HPDE6-C7 (**Figure S14**), energy comparison of defect structures: (i) v_N_{3B}–O_b1‑B_–Cu_b2‑B,B_ with one adsorbed Cu atom vs (ii) v_N_{3B}–O_b1‑B_ with an absorbed Cu–Cu cluster (**Figure S15**), density of states for the nitrogen vacancy defect hosted in 5 × 5 and 6 × 6 BN supercells (**Figure S16**), density of states for the v_N_{3B}–O_b1‑B_–Cu_b2‑B,B_ defect hosted in 5 × 5 and 6 × 6 BN supercells (**Figure S17**), optimized defect structures in CIF format (PDF)
