# Correction to “Enhanced Antibacterial Efficacy of Copper Single-Atom Catalysts on Two-Dimensional Boron Nitride Platform”

**DOI:** 10.1021/acsnano.6c01244

**Published:** 2026-02-25

**Authors:** Wenbo Li, Daniel Maldonado-Lopez, Yingcan Zhao, Cong Wang, Jianxiang Gao, Bowen Sun, Yichao Bai, Linxuan Sun, Mingchuang Zhao, Haoqi He, Jiatao Lou, Qiangmin Yu, Xi Zhang, Vijay Kumar Pandey, Feiyu Kang, Mauricio Terrones, Jose L. Mendoza-Cortes, Yu Lei

In the original paper, there is an error in Figure 3b. In the scanning electron microscopy (SEM) images, the scale bar is incorrectly labeled as “1 nm”. The correct label should be “1 μm”. The corrected Figure 3 is below.

**Figure 3:**
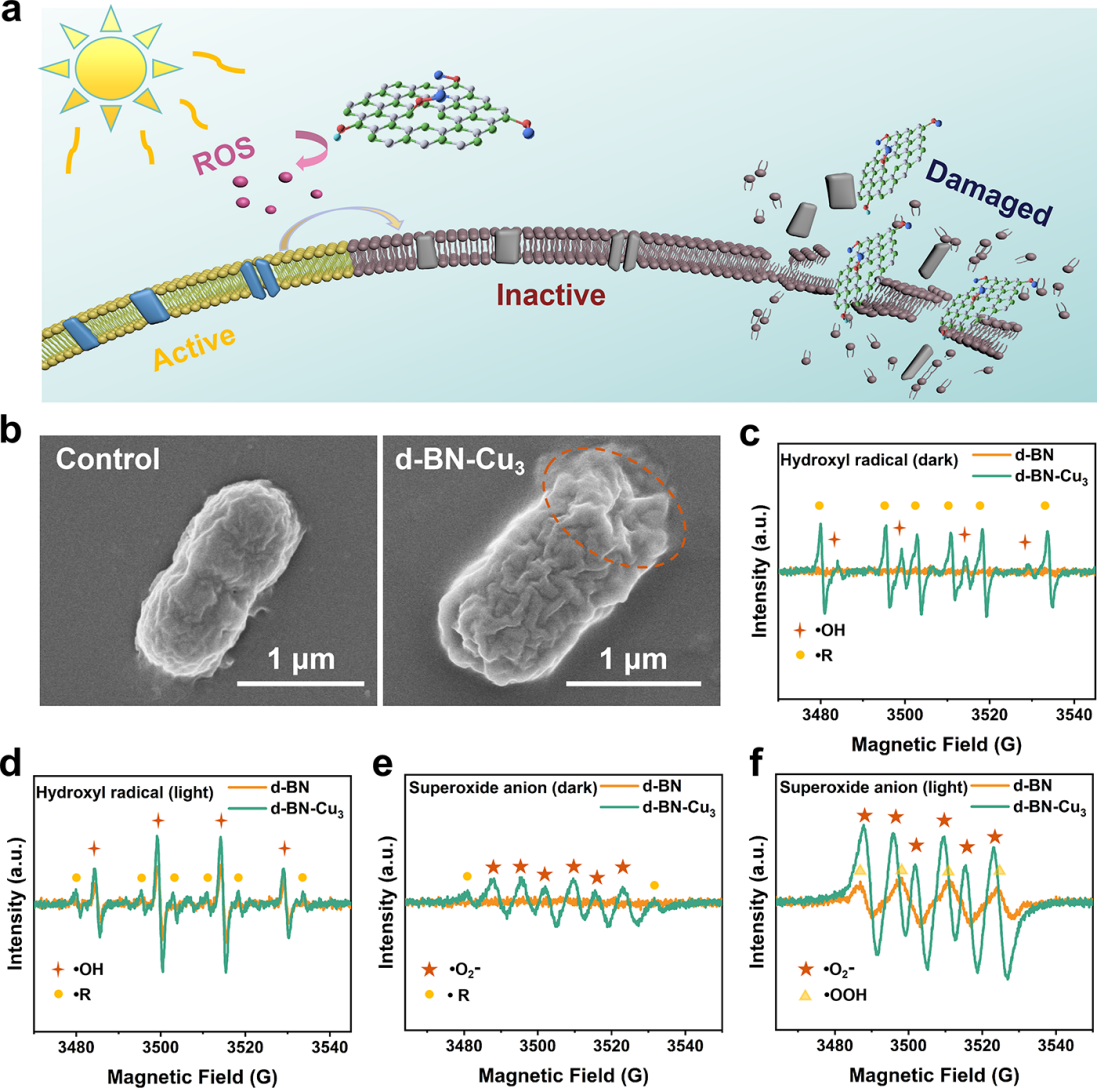
(a) Schematic diagram of the antibacterial mechanism of d-BN-Cu. (b) Morphology of *E. coli* before and after treatment with antibacterial materials under SEM. (c, d) EPR analysis of ROS generation by d-BN-Cu_3_ under illuminated and nonilluminated conditions, showing enhanced •OH production under light exposure. (e, f) Using EPR to test the catalytic effect of d-BN-Cu_3_ on •O_2_^–^ under light and no-light conditions.

In addition, the SEM images in Figure S10 of the Supporting Information (SI) also exhibit the same error. The corrected figure is provided in the revised Supporting Information.

These errors do not affect any experimental data, interpretation, or the scientific conclusions of the work. The intended interpretation of the figures, showing membrane disruption in *E. coli* after treatment with d-BN-Cu_3_, remains unchanged.

All authors have been consulted and approve the submission of this correction.

## Supplementary Material



## Notes

Same description as in the original paper (PDF)

