# Supplementary material for: Correction to “Enhanced Antibacterial Efficacy of Copper Single-Atom Catalysts on Two-Dimensional Boron Nitride Platform”
Source: ACS Nano. 2026 Feb 25;20(9):8068–9. doi: 10.1021/acsnano.6c01244 (PMC12981011; doi:10.1021/acsnano.6c01244)
Supplement: Supplementary file 1 [file nn6c01244_si_001.pdf]

Supporting Information for:

Enhanced Antibacterial Efficacy of Copper Single-Atom Catalysts on Two-Dimensional Boron Nitride Platform

Wenbo Li[1,2#], Daniel Maldonado-Lopez[3#], Yingcan Zhao[4], Cong Wang[1,2], Jianxiang Gao[1,2], Bowen Sun[5], Yichao Bai[1,2], Linxuan Sun[1,2], Mingchuang Zhao[1,2], Haoqi He[1,2], Jiatao Lou[2], Qiangmin Yu[2,6], Xi Zhang[5], Vijay Kumar Pandey[5], Feiyu Kang[1,2,6], Mauricio Terrones[7], Jose L. Mendoza-Cortes[3,8]*, and Yu Lei[1,2]**

[1]Institute of Materials Research, Center of Double Helix, Tsinghua Shenzhen International Graduate School, Tsinghua University, Shenzhen 518055, P. R. China.

[2]Shenzhen Key Laboratory of Advanced Layered Materials for Value-added Applications, Institute of Materials Research, Tsinghua Shenzhen International Graduate School, Tsinghua University, Shenzhen 518055, P. R. China.

[3]Department of Chemical Engineering & Materials Science, Michigan State University, East Lansing, MI 48824, USA.

[4]Environmental Science Program, Department of Life Sciences, Beijing Normal University-Hong Kong Baptist University United International College, Guangdong 519087, P. R. China.

[5]Institute of Biopharmaceutical and Health Engineering, Tsinghua Shenzhen International Graduate School, Tsinghua University, Shenzhen 518055, P.R. China.

[6]Shenzhen Geim Graphene Center, Tsinghua-Berkeley Shenzhen Institute & Shenzhen International Graduate School, Tsinghua University, Shenzhen 518055, P. R. China.

[7]Department of Physics, Department of Chemistry, Department of Materials Science and Engineering, Center for Two-Dimensional and Layered Materials, The Pennsylvania State University, University Park, PA 16802, USA.

[8]Department of Physics & Astronomy, Michigan State University, East Lansing, MI 48824, USA.

*Corresponding authors: leiy07@sz.tsinghua.edu.cn, jmendoza@msu.edu, yingcanzhao@uic.edu.cn

#W.L. and D.M.L. have equal contributions.

The supporting information includes:

1. Instrument Information
2. Chemicals and Reagents
3. Characterization
4. Supporting Results

1. Instrument Information

The size and morphology of materials were characterized using scanning electron microscopy (SEM) (Hitachi SU8010, operating at 5 kV). Aberration-corrected scanning transmission electron microscopy (STEM) (Thermo Fisher Scientific, Spectra 300) was used for element distribution analysis. Atomic force microscopy (AFM) (Oxford Instruments, Cyper ES, MDTC-EQ-M16-01) was employed to measure sample thickness. X-ray photoelectron spectroscopy (XPS) (Thermo Fisher Scientific, PHI Versaprobe 4i, monochromatic Al Ka X-rays, 1484.6 eV) was used for elemental analysis. Inductively Coupled Plasma Emission Spectrometer (ICP-OES) (Spectro Arcos Ⅱ MV) measured metal ion content. Solar simulator (Zolix, Sirius-SS300A-L, 400-1076 nm) provided illumination for photocatalytic antibacterial experiments. Electron Paramagnetic Resonance (EPR) spectroscopy (Bruker, EMXplus-10/12) was conducted to detect radical species. Xenon lamp (LOT-QuantumDesign GmbH, 100 W, 200-2000 nm) provided light for detection of active oxygen. Infrared camera (FOTRIC 220S) was used to record changes in system temperature during photocatalytic antibacterial experiment.

2. Chemicals and Reagents

Hexagonal boron nitride (h-BN, ≤10 μm), anhydrous copper sulfate ($CuSO_4$), cobalt chloride hexahydrate ($CoCl_2{\cdot}6H_2O$), chromium chloride hexahydrate ($CrCl_3{\cdot}6H_2O$), methanol (CH_3OH), glutaraldehyde ($(CH_2O)_2$), and sodium chloride (NaCl) were sourced from Aladdin Chemical Co. Ltd. (Shanghai, China). Ferric chloride hexahydrate ($FeCl_3{\cdot}6H_2O$), manganese chloride tetrahydrate ($MnCl_2{\cdot}4H_2O$), nickel chloride hexahydrate ($NiCl_2{\cdot}6H_2O$), and zinc chloride ($ZnCl_2$) were purchased from Macklin Biochemical Technology Co. Ltd. (Shanghai, China). 5,5-Dimethyl-1-pyrroline N-oxide (DMPO) and 2,2,6,6-Tetramethyl-4-piperidone hydrochloride (TEMP) were purchased from Tongren Institute of Chemical Research. Luria-Bertani (LB) and 4% paraformaldehyde solution were purchased from Solarbio Science & Technology Co. Ltd. (Beijing, China).

3. Characterization

Cu K-edge XAFS analyses were performed with Si (111) crystal monochromators at the BL14W Beam line at the Shanghai Synchrotron Radiation Facility (SSRF) (Shanghai, China). Before the analysis at the beamline, samples were placed into aluminum sample holders and sealed using Kapton tape film. The XAFS spectra were recorded at room temperature using a 4-channel Silicon Drift Detector (SDD) Bruker 5040. Cu K-edge extended X-ray absorption fine structure (EXAFS) spectra were recorded in transmission/fluorescence mode. Negligible changes in the line-shape and peak position of Cu K-edge XANES spectra were observed between two scans taken for a specific sample. The XAFS spectra of these standard samples were recorded in transmission mode. The spectra were processed and analyzed by the software codes Athena.

XPS measurement uses a monochromatic Al K α X-ray source (h ν=1486.6 eV), with a test beam spot size of 200 microns, an electron acceleration voltage of 15 kV, and a test power of 50 W.

Aberration-corrected scanning transmission electron microscopy (STEM) was operated on a cold-field-emission spherical- aberration-corrected transmission electron microscope (Thermo Fisher Spectra 300) operated at 300 kV.

Dilute and disperse the target sample with a 70% ethanol solution, drop it onto a silicon wafer and air dry naturally for SEM and AFM morphology characterization.

4. Supporting Results

Table S1. Cu content determination of BN samples by ICP analysis.

Sample	d-BN	d-BN-Cu_4	d-BN-Cu_3	d-BN-Cu_2	d-BN-Cu_1
Cu content (% at.)	0	0.0089%	0.024%	0.11%	0.26%

Table S2. XPS analysis of material element content (% at.), quantitative analysis of elements in XPS data was performed using the MultiPak software.

Sample/Element	Cu	B	N	O

120BN	0%	44.04%	45.75%	10.22%
d-BN-Cu_4	0.11%	45.78%	47.24%	6.18%
d-BN-Cu_3	0.18%	43.80%	44.61%	11.41%
d-BN-Cu_2	0.38%	44.89%	46.83%	7.91%
d-BN-Cu_1	0.87%	43.14%	45.37%	10.62%

Table S3. Determination of Cu and K ions in the supernatant of the mixture after sterilization by d-BN-Cu_3 using ICP (The sample concentration is 100 ppm).

Element	Cu	K
Content (μg/mL)	0	0.432

Table S4. Statistical summary of EPR signals of hydroxyl radicals in **Figure 3d** (The calculation of peak intensity involves adding the absolute values of the upper and lower peaks of each peak, and including all characteristic peaks belonging to the signal. Normalization basis: Control with light = 100 %).

Sample/Condition	Dark	Light
Control	0%	100%
d-BN	0%	170.96%
d-BN-Cu_3	77.23%	304.65%

Table S5. Statistical summary of EPR signals of hydroxyl radicals in **Figure S12** (The calculation of peak intensity involves adding the absolute values of the upper and lower peaks of each peak, and including all characteristic peaks belonging to the signal. Normalization basis: Control with light = 100 %).

Sample/Condition	Dark	Light
Control	0%	100%

d-BN-Cu_1	172.8%	355.4%
d-BN-Mn	200.4% (•SRO)	173.3% (•SRO)
d-BN-Zn	0%	133.9%
d-BN-Co	0%	90.4%
d-BN-Fe	0%	90.1%
d-BN-Ni	0%	88.2%
d-BN-Cr	0%	58.3%

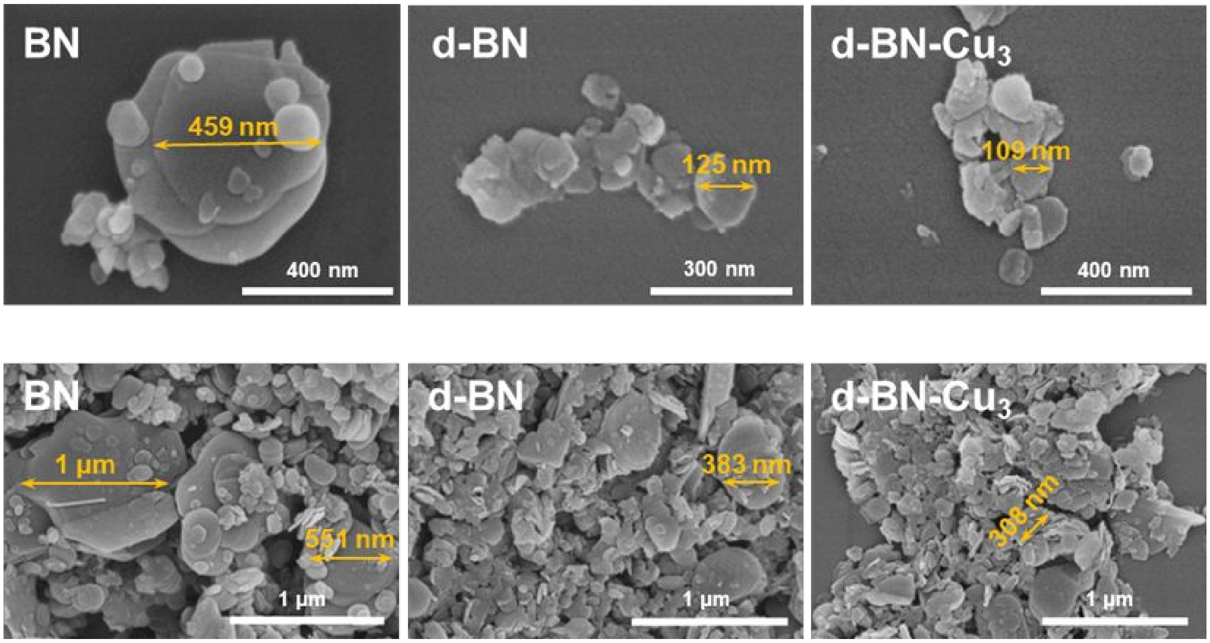

Figure S1. SEM images showing the morphology of BN, defect-engineered BN (d-BN), and copper-loaded d-BN-Cu_3 samples.

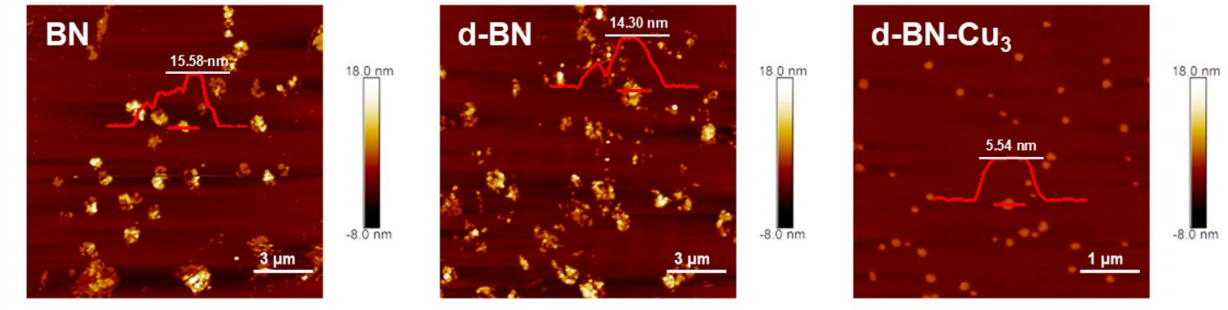

Figure S2. AFM images of BN, d-BN and d-BN-Cu_3.

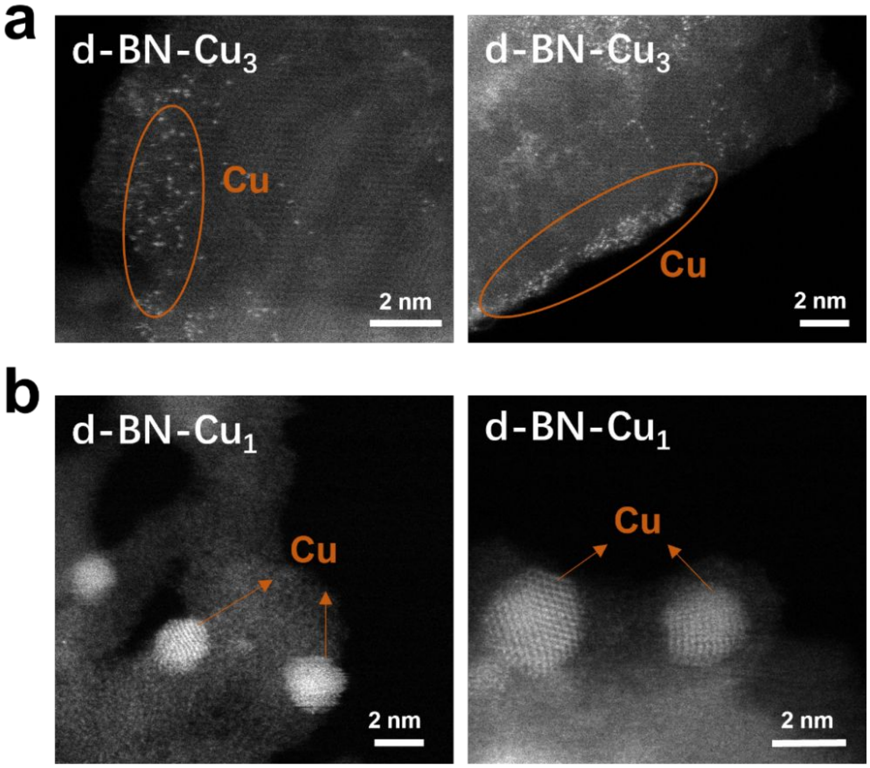

Figure S3. HAADF-STEM images of a) d-BN-Cu_3 and b) d-BN-Cu_1.

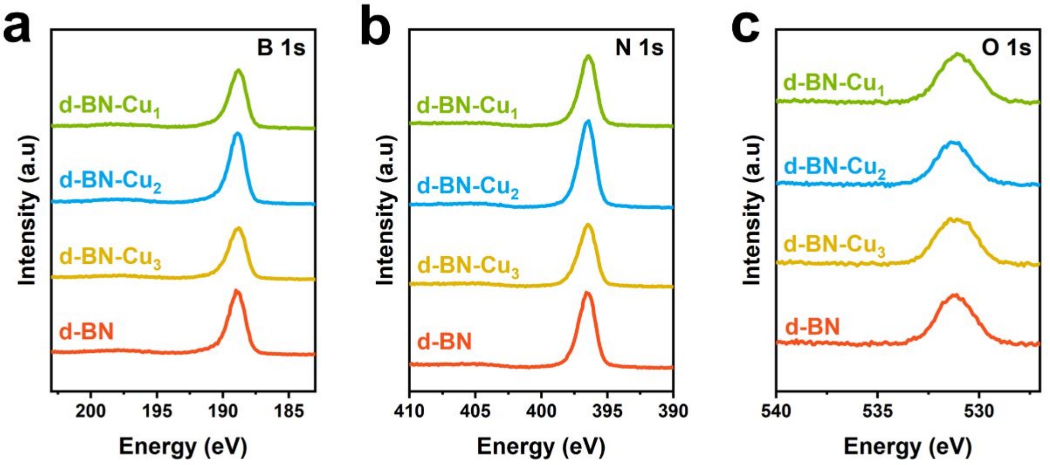

Figure S4. XPS spectra of defect-engineered BN (d-BN) and copper-loaded variants (d-BN-Cu_1, d-BN-Cu_2, and d-BN-Cu_3), showing elemental distributions: (a) boron (B), (b) nitrogen (N), and (c) oxygen (O).

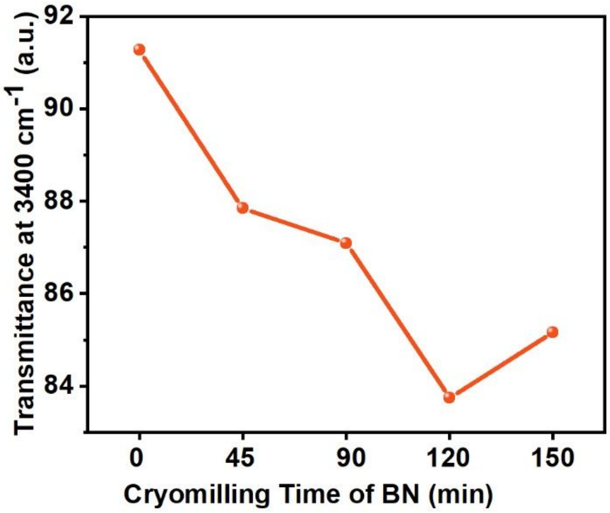

Figure S5. FTIR signals of BN with different cryomilling times were detected, and the transmittance at 3400 cm^{-1} was statistically analyzed.

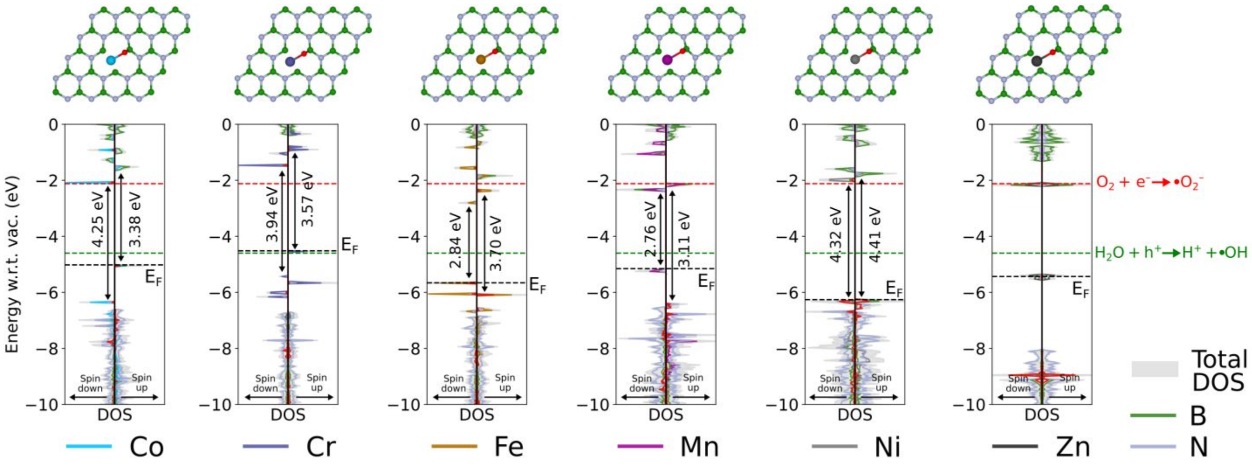

Figure S6. Defect structures hosted in 5×5 supercells incorporating different transition metals in the same configuration as Cu-defective BN structures (i.e. B−O−TM coordination, $\mathbf{v_N\{3B\}{-}O_{b1\text{-}B}{-}TM_{b2\text{-}B,B}}$). Defect geometries were optimized prior to obtaining their properties. In general, these defect structures do not have the correct band alignment/band gap to form both ROS, as opposed to Cu. Further investigation should be carried out to further verify defect stability in this configuration, and if the structures can be tuned to satisfy ROS-generation requirements.

The structures shown in **Figure S6** correspond to $\mathbf{v_N\{3B\}{-}O_{b1\text{-}B}{-}TM_{b2\text{-}B,B}}$ defects (with B−O−TM coordination) hosted in 5×5 lattices, where TM corresponds to first-row transition metals. From left to right the plots correspond to: Co, Cr, Fe, Mn, Ni, and Zn defects. This figure includes a top view of their structure (top) and their corresponding DOS aligned w.r.t. vacuum (bottom). The redox potential levels for the generation of hydroxyl (•OH) and superoxide ($\bullet O_2^-$) radicals are shown as green and red dotted lines, respectively. To photocatalyze the ROS formation reactions, the valence band should fall below the H_2O oxidation reaction potential, and the conduction band above the O_2 reduction reaction potential. Furthermore, structures should have a band gap less than 3.1 eV to fall within the solar irradiation spectrum. The Co, Cr, and Ni structures have adequate band alignment in at least one of their spin channels, but their band gaps are >3.1 eV and prevent photoexcitation within this range. Fe and Mn defects have adequate band gaps in at least one of their spin channels, but their alignments do prohibit the formation of both ROS simultaneously. Interestingly, Zn defects have intermediate energy states that fall at the Fermi level, making this

defect structure conductive. Thus, the electronic structures for these TM defect configurations do not allow for the generation of ROS of interest under solar irradiation conditions. Further work should be carried out to investigate the stability of these TM defects and potential property tuning to achieve appropriate electronic properties to carry out ROS photocatalysis.

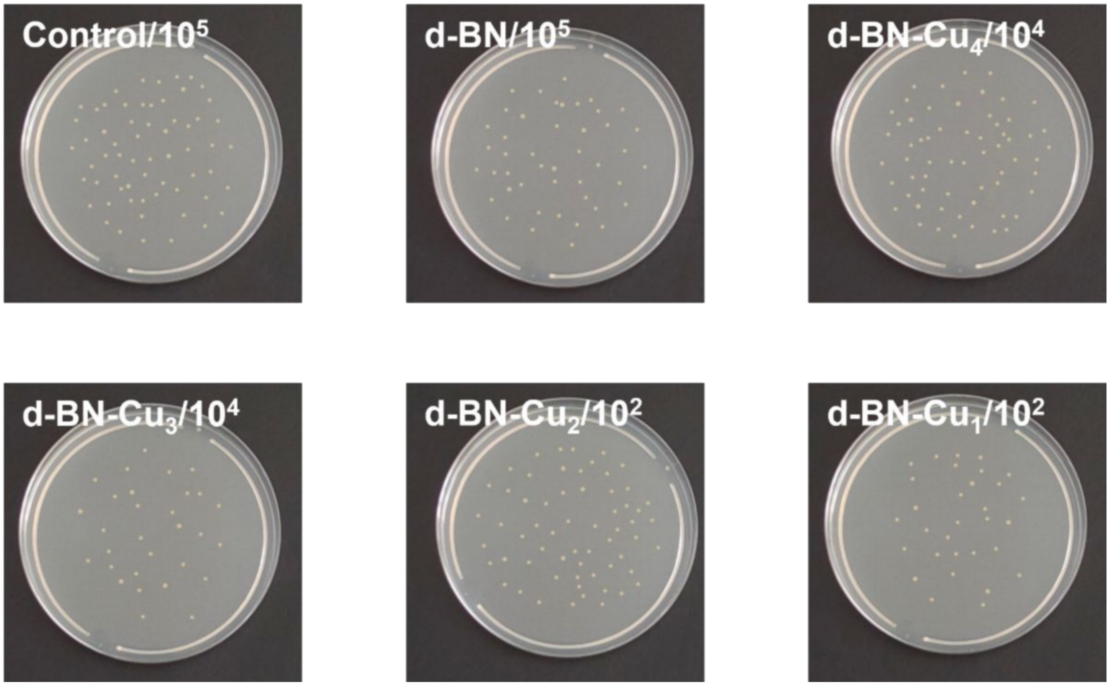

Figure S7. Evaluation of the antibacterial performance of d-BN-Cu (100 ppm) against *S. aureus* using plate colony counting method. The concentration of the bacterial solution was 10^6 CFU mL^{-1}, and it was incubated in a constant temperature shaker (37 ℃/120 rpm) for 3 h. After diluting the control group and d-BN by 10^5 times, the dilution ratio of d-BN-Cu_4 and d-BN-Cu_3 was 10^4, and the dilution ratio of d-BN-Cu_2 and d-BN-Cu_1 was 10^2.

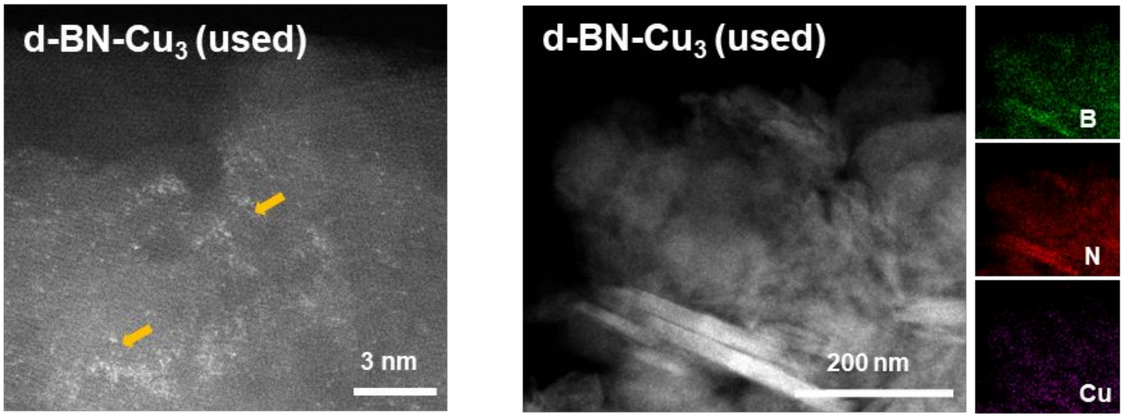

Figure S8. HAADF-TEM image of d-BN-Cu_3 (used) and EDS mapping of d-BN-Cu_3 (used).

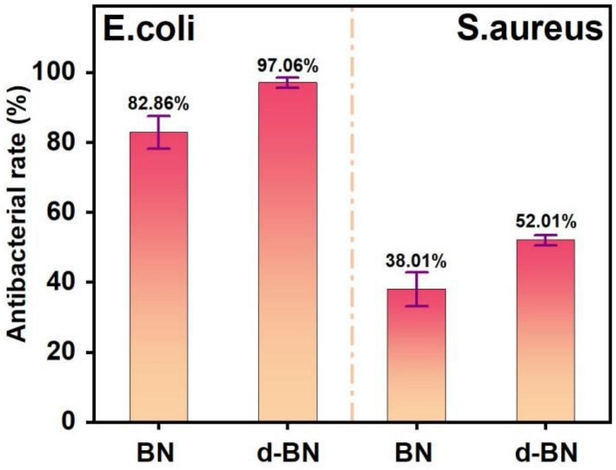

Figure S9. Effect of varying cryomilling times on the antibacterial rate of BN against *E. coli*. Due to the low inherent antibacterial activity of the BN material, a bacterial concentration of 10^4 CFU mL^{-1} was used with deionized water as the solvent.

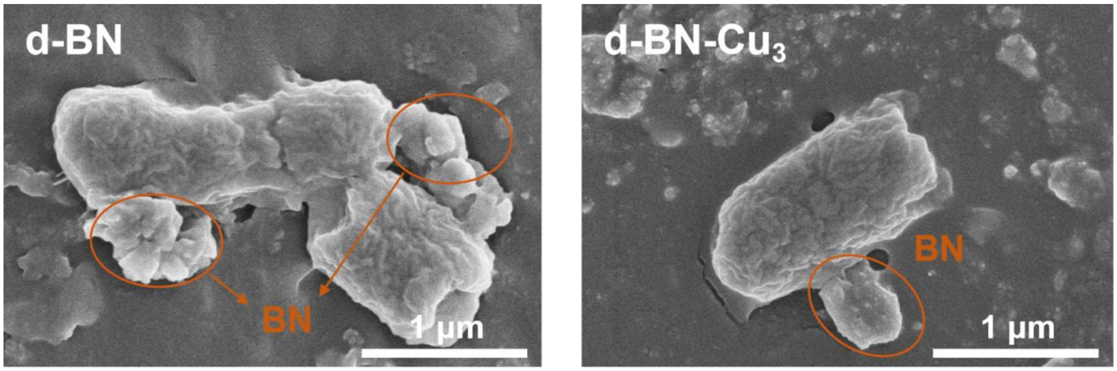

Figure S10. Morphology of *E. coli* before and after treatment with d-BN and d-BN-Cu_3 under SEM.

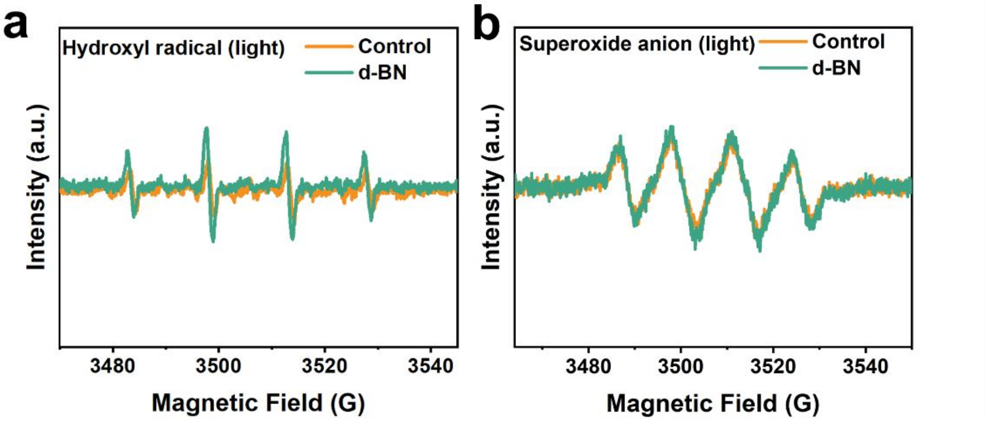

Figure S11. EPR analysis of the catalytic effect of control and d-BN under light: (a) hydroxyl radicals (•OH) and (b) superoxide radicals (•O_2^-). The signals were determined before and after 1 min of light exposure, respectively. DMPO (100 mM) was chosen as the trapping agent and deionized water as the solvent.

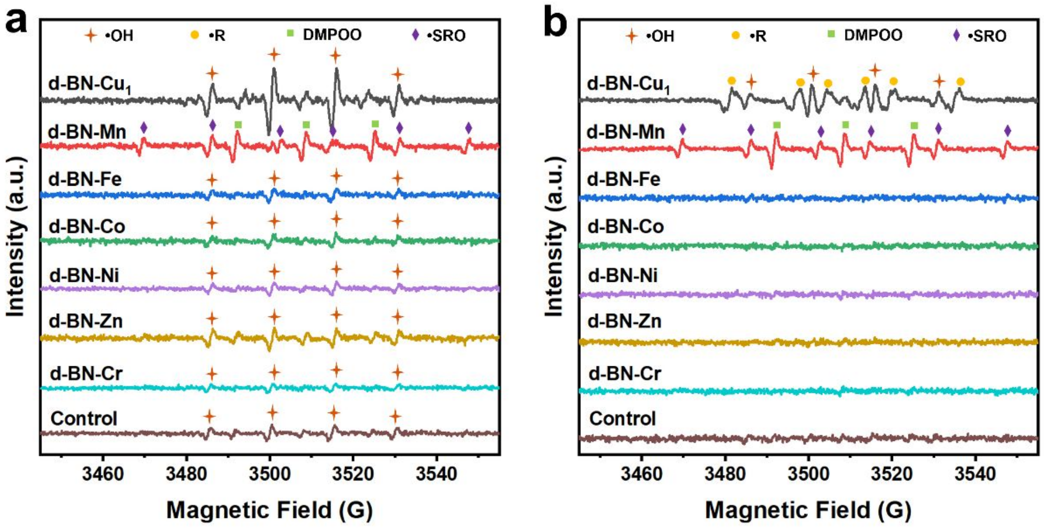

Figure S12. The influence of d-BN with seven different transition group metals (Cu, Mn, Fe, Co, Ni, Zn, Cr) on the EPR signal of hydroxyl radicals under light (a) and dark (b). The signals were determined before and after 1 min of light exposure, respectively. DMPO (100 mM) was chosen as the trapping agent and deionized water as the solvent.

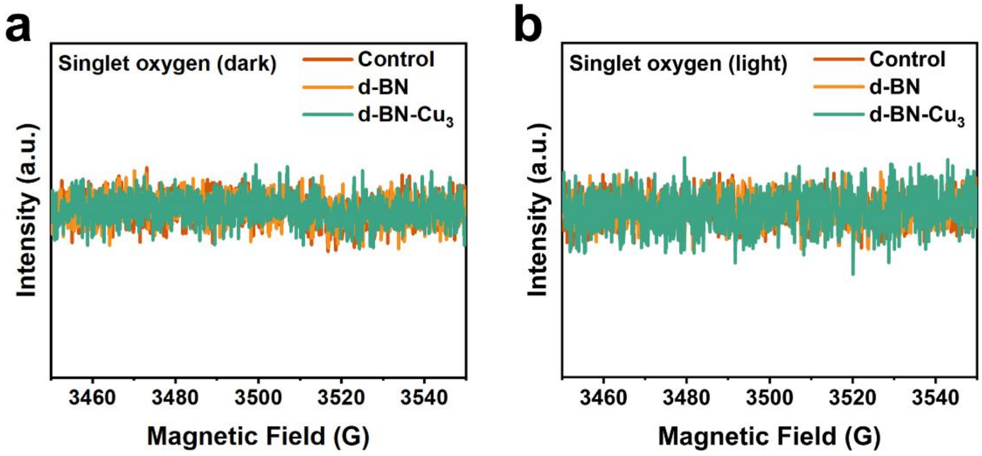

Figure S13. Using EPR to test the catalytic effect of d-BN and d-BN-Cu_3 on singlet oxygen under dark (a) and light (b). TEMP (20 mM) was selected as the capture agent and deionized water was used as the solvent. The signals were determined before and after 1 min of light exposure, respectively.

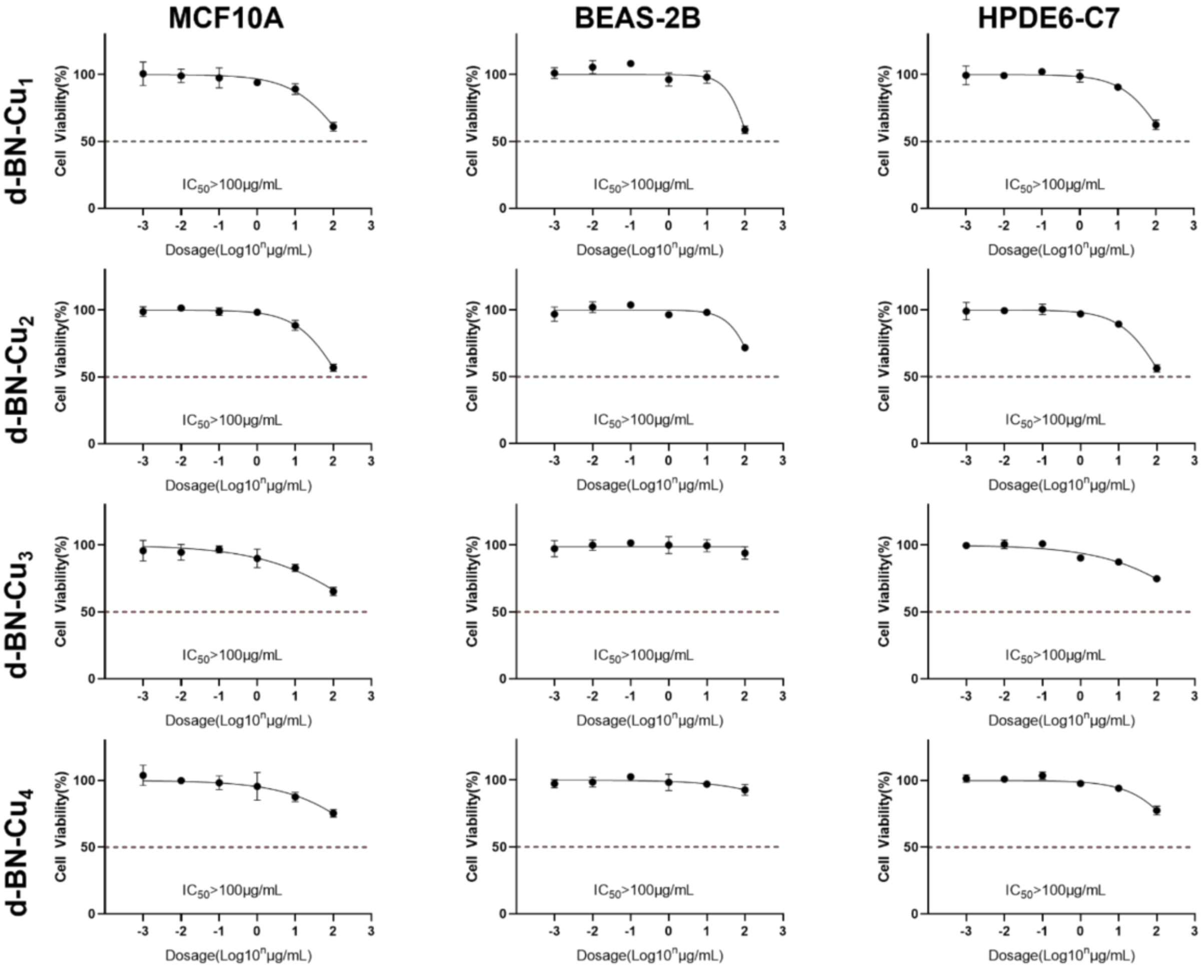

Figure S14. Cytotoxicity assay of d-BN-Cu for MCF10A (normal human epithelial breast cell line), BEAS-2B (normal human bronchial epithelial cell line) and HPDE6-C7 (normal human pancreatic ductal epithelial cells). 5×10^3 cells were plated in a 96-well plate overnight, and the cell viability was measured according to the alamarBlue™ (Invitrogen, DAL1025) instructions after 72 hours of exposure to diluted compounds (0, 0.01, 0.1, 1, 10, 100 μg/mL dissolved in saline) in the medium.

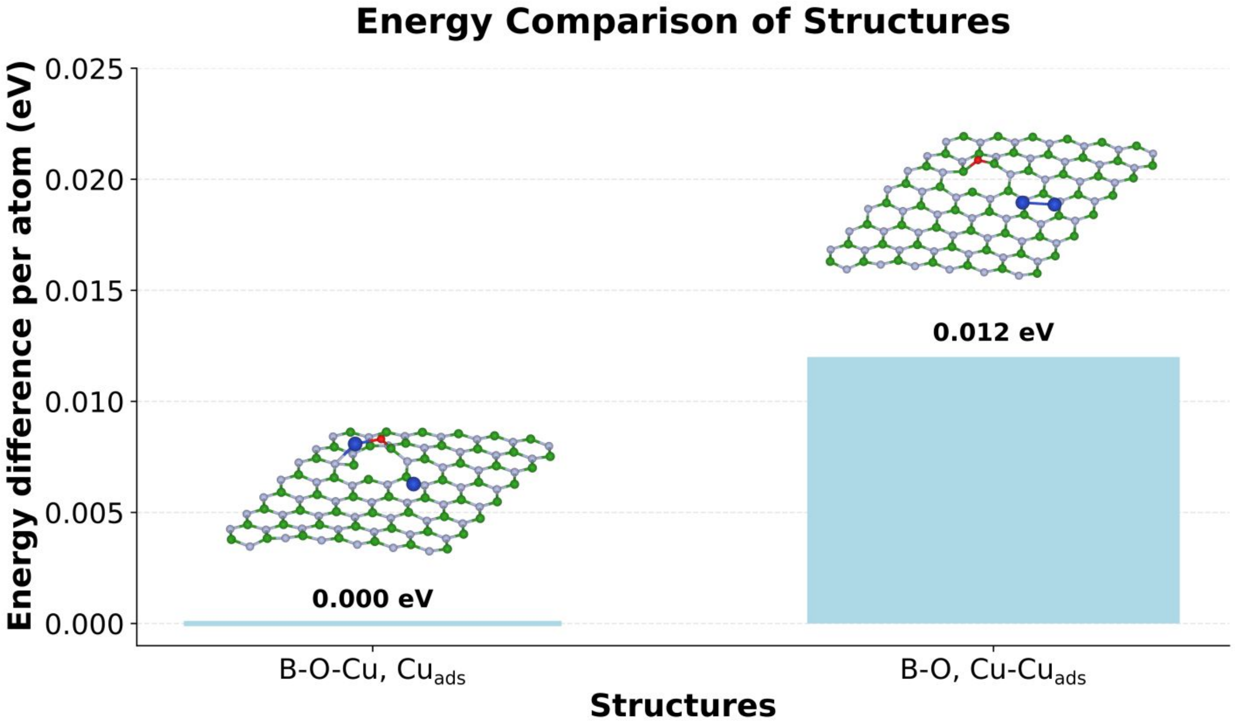

Figure S15. Bar plot energy comparison of a (left) BN defect structure with B−O−Cu coordination and an adsorbed Cu atom vs. (right) a structure with B−O coordination and an adsorbed Cu−Cu cluster. These defects were hosted on a 6×6 supercell. The structure with B−O−Cu coordination shows higher stability by 0.012 eV/atom, suggesting that Cu atoms preferentially bind with the defective sites in the presence of oxygen.

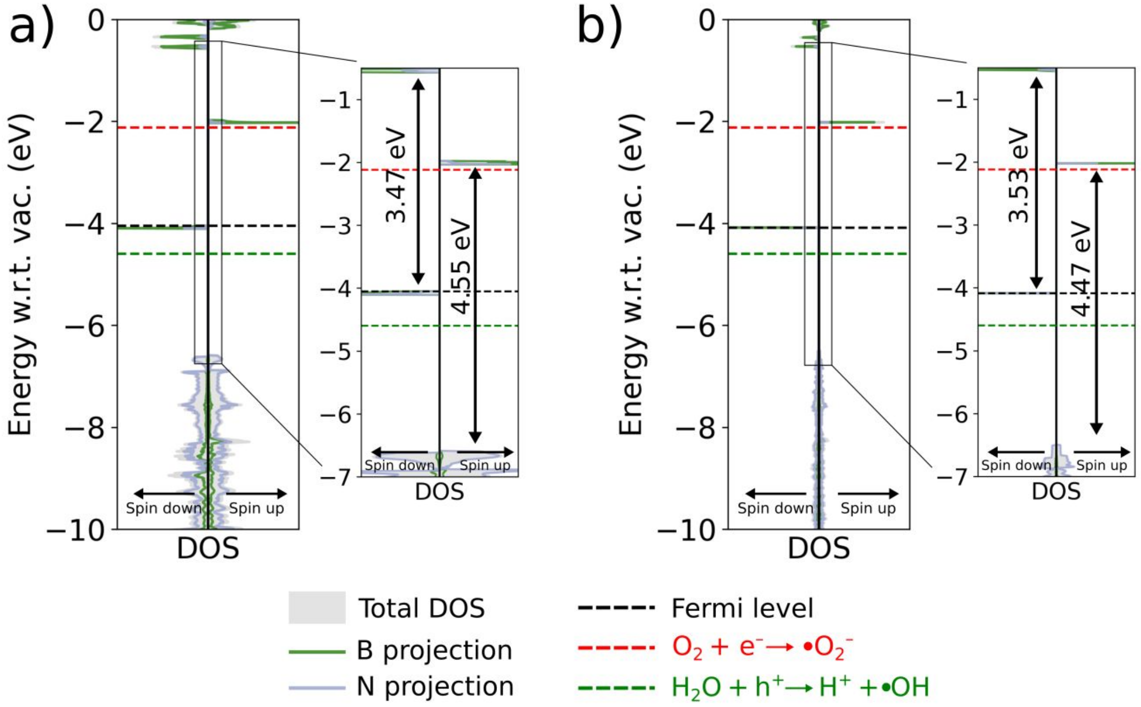

Figure S16. Atom-projected, spin-polarized density of states for the nitrogen vacancy defect hosted in (a) 5×5 and (b) 6×6 BN supercells. The inset shows a zoom into the region around -7 to -0.5 eV w.r.t. vacuum. Compared to the 4×4 supercell, the intermediate defect levels are organized in narrower bands, which is attributed to the lower defect density. However, in general, the DOS profile (band edges, band gaps, and consequently its catalytic properties) are similar to the 4×4 supercell structure.

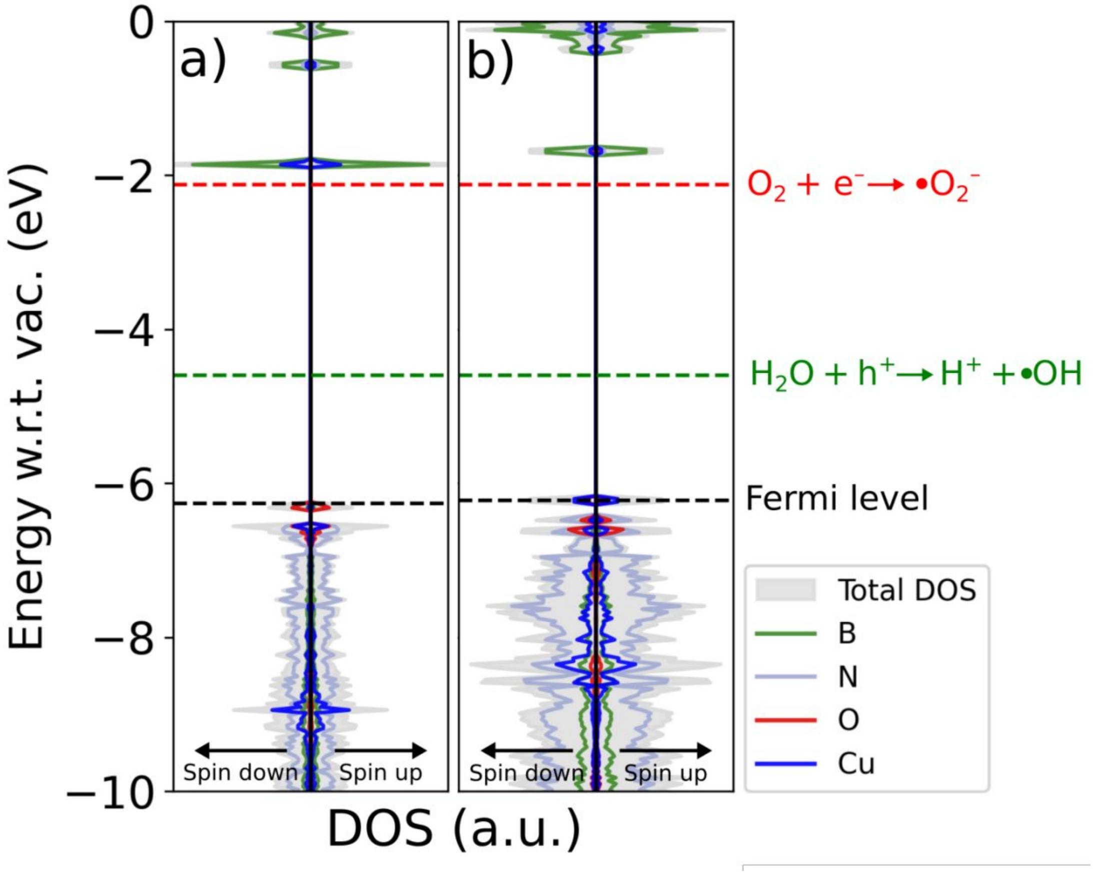

Figure S17. Atom-projected, spin-polarized density of states for the $v_N\{3B\}-O_{b1\text{-}B}-Cu_{b2\text{-}B,B}$ defect hosted in (a) 5x5 (band gap 4.40 eV) and (b) 6x6 (band gap 4.54 eV) BN supercells. The main difference when reducing the defect density (i.e., making the supercell larger) is a reduction in the number of intermediate states around -2 eV. These results predict that the fundamental electronic structure leading to the photocatalytic activity of the $v_N\{3B\}-O_{b1\text{-}B}-Cu_{b2\text{-}B,B}$ defect does not drastically change with defect density.

Optimized Computational Defect Structures (CIF format)

CIF files provided for pristine BN, defective BN, and Cu-doped BN, and multilayer structure of interest. Note that slab CIF files have a **c** *lattice parameter of 40 Å for better visualization in the VESTA software. However, calculations were carried out with a cell length of 500 Å in the c-direction to avoid interactions between periodic images of the slab. This spacing is automatically assigned by the CRYSTAL23 software when setting up slab calculations. All structures were fully optimized (lattice parameters and atom positions).*

Pristine BN slab

```
data_hBN_slab_pristine

_cell_length_a 2.49403162
_cell_length_b 2.49403162
_cell_length_c 40
_cell_angle_alpha 90.000000
_cell_angle_beta 90.000000
_cell_angle_gamma 120.000000
_symmetry_space_group_name_H-M        'P 1'
_symmetry_Int_Tables_number          1

loop_
_symmetry_equiv_pos_as_xyz
   'x, y, z'

loop_
_atom_site_label
_atom_site_type_symbol
_atom_site_fract_x
_atom_site_fract_y
_atom_site_fract_z
B001 B -3.333333333333E-01 3.333333333333E-01 0.0
N002 N 3.333333333333E-01 -3.333333333333E-01 0.0
```

Nitrogen vacancy (v_N) hosted in a 4x4 BN supercell

```
data_v_N_4x4_slab

_cell_length_a 9.86798744
_cell_length_b 9.79027343
_cell_length_c 40
_cell_angle_alpha 90.000000
_cell_angle_beta 90.000000
_cell_angle_gamma 119.732000
_symmetry_space_group_name_H-M        'P 1'
_symmetry_Int_Tables_number          1
```

```
loop_
_symmetry_equiv_pos_as_xyz
   'x, y, z'

loop_
_atom_site_label
_atom_site_type_symbol
_atom_site_fract_x
_atom_site_fract_y
_atom_site_fract_z
B001 B 1.630109379074E-01 1.045489105894E-01 0.99598515646425
B002 B 1.633285449980E-01 3.088224110227E-01 0.9958529667137499
N003 N 8.325885740826E-02 4.035337877745E-01 0.9938336756692501
B004 B 1.652314223896E-01 -4.234154709735E-01 0.9948794652197501
N005 N 8.411845285508E-02 -3.329302458917E-01 0.9939474337255
B006 B 1.652108725574E-01 -1.613056883253E-01 0.994901662621
N007 N 8.322423495543E-02 -7.026792682857E-02 0.9938343411050001
B008 B 4.140528657108E-01 8.424357178160E-02 1.001177695978
N009 N 3.297585683737E-01 1.686204207949E-01 1.00183985950475
B010 B 4.200344669377E-01 3.350127491771E-01 1.0025034657085
N011 N 3.299633456317E-01 4.113351215401E-01 1.00165272959825
B012 B 4.141536242301E-01 -4.200566039945E-01 1.0010290639555
N013 N 3.313978810018E-01 -3.359245177116E-01 0.9980058504672501
B014 B 4.143164418014E-01 -1.678033715163E-01 0.9999870717715
N015 N 3.313608975770E-01 -8.261626668322E-02 0.9980970999752501
B016 B -3.330445561520E-01 8.296832822758E-02 1.0042116896475
N017 N -4.175545475542E-01 1.663919646756E-01 1.0027019713154999
B018 B -3.309835384622E-01 3.337324996482E-01 1.00126361446475
N019 N -4.137732834934E-01 4.180999977945E-01 1.002295451383
B020 B -3.310100724003E-01 -4.147762139571E-01 1.00116817580125
N021 N -4.175248836638E-01 -3.339237342345E-01 1.00254943860025
B022 B -3.330930879362E-01 -1.660542351068E-01 1.004105765536
N023 N -4.188032900624E-01 -8.439477753812E-02 1.00405903429325
B024 B -7.712543479947E-02 8.626993359390E-02 1.00953518218475
N025 N -1.639844230353E-01 1.663236011917E-01 1.005644645315
B026 B -8.156996460571E-02 3.309360559937E-01 0.9988483474580001
N027 N -1.646679860416E-01 4.160234382152E-01 0.9979839009175
B028 B -8.217183159484E-02 -4.161323754729E-01 0.99591829237075
N029 N -1.647080120707E-01 -3.308027290512E-01 0.9979020522705
B030 B -8.163066984162E-02 -1.626438008442E-01 0.9987715321894999
N031 N -1.641088326218E-01 -8.048183389117E-02 1.00551336777475
```

Nitrogen vacancy (v_N) hosted in a 5x5 BN supercell

```
data_v_N_5x5_SLAB_opt_DZ.out

_cell_length_a 12.37264512
_cell_length_b 12.30999225
_cell_length_c 40
_cell_angle_alpha 90.000000
_cell_angle_beta 90.000000
_cell_angle_gamma 119.829989
_symmetry_space_group_name_H-M       'P 1'
_symmetry_Int_Tables_number          1
```

```
loop_
_symmetry_equiv_pos_as_xyz
  'x, y, z'

loop_
_atom_site_label
_atom_site_type_symbol
_atom_site_fract_x
_atom_site_fract_y
_atom_site_fract_z
B001 B 1.304680273857E-01 8.411453725208E-02 0.99618733255975
B002 B 1.306265174917E-01 2.465308970468E-01 0.99608643329075
N003 N 6.641305487847E-02 3.215572587724E-01 0.99390590779
B004 B 1.322386010967E-01 4.592004232630E-01 0.9945730688447499
N005 N 6.707378479516E-02 -4.696413140996E-01 0.99370063951675
B006 B 1.325817263125E-01 -3.337010385579E-01 0.9942869103482501
N007 N 6.707305047856E-02 -2.632769051648E-01 0.9936984276652501
B008 B 1.322282827750E-01 -1.269437385001E-01 0.994608829363
N009 N 6.639876020095E-02 -5.514146603211E-02 0.993915049708
B010 B 3.309441286825E-01 6.787311615376E-02 1.00100763889
N011 N 2.636084528733E-01 1.350875799397E-01 1.0021544705755
B012 B 3.355707605030E-01 2.677911941120E-01 1.0032303768655
N013 N 2.637190976484E-01 3.286331337702E-01 1.00200628893
B014 B 3.310136256872E-01 4.631546768968E-01 1.00085916955375
N015 N 2.652385506800E-01 -4.697368687290E-01 0.9971903342399999
B016 B 3.317984135289E-01 -3.351347839200E-01 0.9982154848145001
N017 N 2.657751981834E-01 -2.670925181821E-01 0.996236643918
B018 B 3.317841701151E-01 -1.330368260674E-01 0.9982815227199999
N019 N 2.652110867028E-01 -6.500130141216E-02 0.9972992642430001
B020 B -4.672367397221E-01 6.670154133537E-02 1.00253449893
N021 N 4.653610735780E-01 1.334150923936E-01 1.00268159656225
B022 B -4.653655258583E-01 2.670830337908E-01 1.00290263875725
N023 N 4.684055482418E-01 3.342076424970E-01 1.003913660397
B024 B -4.653670950229E-01 4.675470407134E-01 1.00280586183025
N025 N 4.653986746009E-01 -4.680175885342E-01 1.002522121922
B026 B -4.672275731802E-01 -3.339205693085E-01 1.0023946950195
N027 N 4.651433931407E-01 -2.677429663476E-01 1.00148401981725
B028 B -4.678136072732E-01 -1.338994709740E-01 1.00299988122725
N029 N 4.651288019795E-01 -6.709852647217E-02 1.00156954382525
B030 B -2.655844623235E-01 6.647360431465E-02 1.0051061732805
N031 N -3.329988466693E-01 1.328997972577E-01 1.00310951604575
B032 B -2.652251906590E-01 2.664473336285E-01 1.0008056560505
N033 N -3.319321209016E-01 3.335745413892E-01 1.00115002533075
B034 B -2.652222113402E-01 4.673735054005E-01 0.9993395131087499
N035 N -3.319433786843E-01 -4.655317996078E-01 1.00106043869025
B036 B -2.652488827557E-01 -3.317067445869E-01 1.00068467190275
N037 N -3.330141002523E-01 -2.659172038280E-01 1.00296309481575
B038 B -2.656286443039E-01 -1.321082267458E-01 1.005017668696
N039 N -3.340213533822E-01 -6.701774041096E-02 1.00580618684425
B040 B -6.138146763383E-02 6.921621393582E-02 1.009890078557
N041 N -1.306366183035E-01 1.330884615167E-01 1.0060479179602502
B042 B -6.534731444666E-02 2.641850931072E-01 0.9989840327525
N043 N -1.320852444277E-01 3.318435406841E-01 0.9977756143604999
B044 B -6.605479262290E-02 4.656201677321E-01 0.995285500684
N045 N -1.322638581413E-01 -4.661513498201E-01 0.995920687818
B046 B -6.605913207061E-02 -3.317143110472E-01 0.9952475257942501
```

```
N047 N -1.321127931300E-01 -2.639823501312E-01 0.9976805730124999
B048 B -6.538325198458E-02 -1.296035445420E-01 0.998914441236
N049 N -1.307155764703E-01 -6.383327388208E-02 1.00595837093425
```

Nitrogen vacancy (v_N) hosted in a 6x6 BN supercell

```
data_v_N_6x6_slab

_cell_length_a                        14.81980844
_cell_length_b                        14.87250135
_cell_length_c                        40.00000000
_cell_angle_alpha                      90.000000
_cell_angle_beta                       90.000000
_cell_angle_gamma                      119.883258
_symmetry_space_group_name_H-M           'P 1'
_symmetry_Int_Tables_number             1

loop_
_symmetry_equiv_pos_as_xyz
   'x, y, z'

loop_
   _atom_site_label
   _atom_site_type_symbol
   _atom_site_fract_x
   _atom_site_fract_y
   _atom_site_fract_z
 B001   B  -0.4036139435  -0.4421766693  0.2536362400
 B002   B  -0.3911592376  -0.2823046994  0.2398032918
 N003   N  -0.4442479492  -0.2246794882  0.2436553185
 B004   B  -0.3888210319  -0.1123687548  0.2442976657
 N005   N  -0.4440463369  -0.0562632515  0.2465021138
 B006   B  -0.3887793281  0.0554138506  0.2466567988
 N007   N  -0.4442944954  0.1113807419  0.2472607700
 B008   B  -0.3891259783  0.2230320343  0.2479533512
 N009   N  -0.4446314681  0.2791142846  0.2470480091
 B010   B  -0.3901457698  0.3909832545  0.2489382072
 N011   N  -0.4459996957  0.4470459083  0.2475833830
 B012   B  -0.2284236440  -0.4435484470  0.2555053069
 N013   N  -0.2878737502  -0.3886229215  0.2559603734
 B014   B  -0.2256969320  -0.2788731947  0.2508939490
 N015   N  -0.2804424325  -0.2246869217  0.2436626809
 B016   B  -0.2235545031  -0.1123724231  0.2442948600
 N017   N  -0.2777806235  -0.0555600658  0.2431654458
 B018   B  -0.2220227212  0.0559560530  0.2452546555
 N019   N  -0.2774060445  0.1118807203  0.2464439690
 B020   B  -0.2220373252  0.2231770527  0.2488797790
 N021   N  -0.2778472445  0.2789700282  0.2497612338
 B022   B  -0.2231904166  0.3900825052  0.2525764470
 N023   N  -0.2797560358  0.4456341470  0.2530269648
 B024   B  -0.0571863264  -0.4439311137  0.2562213554
 N025   N  -0.1151939797  -0.3891266804  0.2564972959
 B026   B  -0.0576435472  -0.2781751813  0.2550015061
```

```
N027   N  -0.1138290484  -0.2231517477  0.2523183300
B028   B  -0.0571337210  -0.1122035854  0.2494022056
N029   N  -0.1122222412  -0.0562662119  0.2465029400
B030   B  -0.0558084589  0.0554131904  0.2466554950
N031   N  -0.1107136213  0.1118806598  0.2464463049
B032   B  -0.0547840977  0.2231781922  0.2488820203
N033   N  -0.1105841880  0.2788304827  0.2504221743
B034   B  -0.0550845251  0.3898293215  0.2533746843
N035   N  -0.1117414233  0.4450305590  0.2546940646
B036   B  0.1132576621  -0.4439301502  0.2562233019
N037   N  0.0554276975  -0.3891456260  0.2567894390
B038   B  0.1109498924  -0.2780982935  0.2557750922
N039   N  0.0543362950  -0.2229239052  0.2547903626
B040   B  0.1101931027  -0.1120363187  0.2518319607
N041   N  0.0544165089  -0.0564828105  0.2497105622
B042   B  0.1103908860  0.0547241805  0.2478444168
N043   N  0.0556752078  0.1113812180  0.2472679474
B044   B  0.1121611709  0.2230344755  0.2479560151
N045   N  0.0568200059  0.2789720128  0.2497679106
B046   B  0.1132766311  0.3900846212  0.2525799257
N047   N  0.0567743699  0.4450316719  0.2546985504
B048   B  0.2848802897  -0.4435457448  0.2555121027
N049   N  0.2260695493  -0.3891253235  0.2564988477
B050   B  0.2794672531  -0.2781737455  0.2549961952
N051   N  0.2227382463  -0.2229229496  0.2547869322
B052   B  0.2777684790  -0.1120357631  0.2518290251
N053   N  0.2217402781  -0.0565177552  0.2506603100
B054   B  0.2772526892  0.0545060000  0.2480009255
N055   N  0.2216459197  0.1102776165  0.2469251016
B056   B  0.2773608268  0.2215216727  0.2459881607
N057   N  0.2237515569  0.2791181253  0.2470450294
B058   B  0.2811369776  0.3909896213  0.2489350085
N059   N  0.2253944241  0.4456365451  0.2530241385
B060   B  0.4614583678  -0.4421602804  0.2536296052
N061   N  0.3992545641  -0.3886175449  0.2559610481
B062   B  0.4468204568  -0.2788699062  0.2508873393
N063   N  0.3906748842  -0.2231485722  0.2523134845
B064   B  0.4449279297  -0.1122014907  0.2493955940
N065   N  0.3890990071  -0.0564811518  0.2497030993
B066   B  0.4443321022  0.0547248345  0.2478381938
N067   N  0.3886316000  0.1102775348  0.2469228775
B068   B  0.4441617560  0.2215212694  0.2459865506
N069   N  0.3883339171  0.2766660730  0.2449816127
B070   B  0.4436300178  0.3872603179  0.2461842873
N071   N  0.3930555631  0.4470569128  0.2475798553
```

Nitrogen vacancy with Oxygen (v_N{3B}−$O_{b1\text{-}B}$) hosted in a 4x4 BN supercell

```
data_v_N-O_4x4_slab

_cell_length_a 9.93779707
_cell_length_b 9.93764890
_cell_length_c 40
_cell_angle_alpha 90.000000
```

```
_cell_angle_beta 90.000000
_cell_angle_gamma 121.376900
_symmetry_space_group_name_H-M        'P 1'
_symmetry_Int_Tables_number          1

loop_
_symmetry_equiv_pos_as_xyz
   'x, y, z'

loop_
_atom_site_label
_atom_site_type_symbol
_atom_site_fract_x
_atom_site_fract_y
_atom_site_fract_z
B001 B 1.811194566083E-01 6.884855386580E-02 0.979707716476
B002 B 1.405073802302E-01 3.093264948605E-01 1.0050075582782498
N003 N 8.054406556817E-02 4.137521842210E-01 1.006827003265
B004 B 1.666730815134E-01 -4.191846708424E-01 1.0042993745367501
N005 N 8.481192990934E-02 -3.348654799514E-01 1.003465584675
B006 B 1.696753762465E-01 -1.703406061360E-01 0.997081653199
N007 N 8.662995687298E-02 -8.901645872345E-02 0.99192668744475
B008 B 4.203379684655E-01 8.031745529993E-02 0.9970619904235001
N009 N 3.389947925863E-01 1.633523834759E-01 0.9919164424490001
B010 B 4.108471544658E-01 3.268483093713E-01 0.99964854095275
N011 N 3.165946694306E-01 3.986173658505E-01 1.002996507715
B012 B 4.110564072566E-01 -4.264269001423E-01 1.0032182443789999
N013 N 3.338563550117E-01 -3.383070753060E-01 1.00239250031375
B014 B 4.201405248317E-01 -1.701519316100E-01 1.000251786144
N015 N 3.377256065344E-01 -8.773618881270E-02 0.9960500620195001
B016 B -3.308230298450E-01 8.332279896508E-02 1.00428164568875
N017 N -4.151309760173E-01 1.651909369299E-01 1.00343148683225
B018 B -3.362736503061E-01 3.328724891641E-01 1.0052243837155
N019 N -4.208548502751E-01 4.142817754144E-01 1.0045570116205
B020 B -3.335846633812E-01 -4.164237071914E-01 1.004845823354
N021 N -4.160699894638E-01 -3.339240346311E-01 1.003546880695
B022 B -3.235648530725E-01 -1.610467219255E-01 1.00321036373825
N023 N -4.117053936847E-01 -8.386370599659E-02 1.00238546823375
B024 B -5.933120930213E-02 1.095101182284E-01 1.0050048645482499
N025 N -1.637677180926E-01 1.694480703575E-01 1.006808098946
B026 B -8.649544015894E-02 3.364820750135E-01 1.00740159363175
N027 N -1.687496841118E-01 4.187402104083E-01 1.00752602411975
B028 B -8.287068363111E-02 -4.137286953883E-01 1.0052547750875
N029 N -1.642865186349E-01 -3.291532279449E-01 1.0045789517847499
B030 B -7.684641894826E-02 -1.608554673626E-01 0.9996695736950001
N031 N -1.486080945124E-01 -6.657864281624E-02 1.0030022031525
O032 O 1.336720722498E-01 1.164284002765E-01 0.9524191988857501
```

Nitrogen vacancy with Copper (v_N{3B}−$Cu_{b1\text{-}B}$) hosted in a 4x4 BN supercell

```
data_hBN_v_N-Cu_4x4_slab

_cell_length_a 9.92217862
_cell_length_b 9.92215205
```

```
_cell_length_c 40
_cell_angle_alpha 90.000000
_cell_angle_beta 90.000000
_cell_angle_gamma 121.129100
_symmetry_space_group_name_H-M        'P 1'
_symmetry_Int_Tables_number          1

loop_
_symmetry_equiv_pos_as_xyz
   'x, y, z'

loop_
_atom_site_label
_atom_site_fract_x
_atom_site_fract_y
_atom_site_fract_z
B001 B 1.726536100897E-01 7.732262827710E-02 0.9830344788415
Cu002 Cu 7.842998574663E-02 1.715865213555E-01 0.954738545749
B003 B 1.394861965368E-01 3.075942961731E-01 1.00575788293075
N004 N 7.925491738993E-02 4.138600284283E-01 1.0029488500815
B005 B 1.653836522642E-01 -4.188517713808E-01 1.002288761465
N006 N 8.356249832152E-02 -3.338889749776E-01 1.001011710508
B007 B 1.680442030329E-01 -1.677253290972E-01 0.99671336558125
N008 N 8.480491730195E-02 -8.497791967961E-02 0.9924466986415
B009 B 4.177260961900E-01 8.195638344393E-02 0.996701237944
N010 N 3.349716290629E-01 1.651904398456E-01 0.99243272196325
B011 B 4.102920426520E-01 3.283578307005E-01 1.0011366571262499
N012 N 3.174887260847E-01 4.003259187930E-01 1.0058402465405
B013 B 4.103438456815E-01 -4.253844122049E-01 1.0053616965902499
N014 N 3.329328230665E-01 -3.369287703084E-01 1.00296190767825
B015 B 4.187448993164E-01 -1.687414500388E-01 1.00087380352975
N016 N 3.359190370868E-01 -8.591975252897E-02 0.99617480966825
B017 B -3.311381137285E-01 8.461999529110E-02 1.0022705335365
N018 N -4.161015341722E-01 1.664405640340E-01 1.00098973604325
B019 B -3.367393210979E-01 3.339472138522E-01 1.002868552097
N020 N -4.208781748939E-01 4.155522824880E-01 1.004166048228
B021 B -3.342349417912E-01 -4.157721804132E-01 1.00557488170625
N022 N -4.170058351290E-01 -3.329948292227E-01 1.00589150335375
B023 B -3.246152078554E-01 -1.603441512960E-01 1.005359550913
N024 N -4.130650151207E-01 -8.293000779168E-02 1.00295559841125
B025 B -5.758478050797E-02 1.105132899850E-01 1.0057470324375
N026 N -1.638501695297E-01 1.707469321499E-01 1.002927991243
B027 B -8.715183925510E-02 3.371518983804E-01 1.003314727331
N028 N -1.694738752622E-01 4.194735946614E-01 1.0034627107077498
B029 B -8.394701514379E-02 -4.132591186657E-01 1.00288176989925
N030 N -1.655574900407E-01 -3.291273845641E-01 1.0041777875859998
B031 B -7.836569432357E-02 -1.603008246791E-01 1.00114650859475
N032 N -1.503300719723E-01 -6.749294101035E-02 1.00584169307275
```

Nitrogen vacancy with Oxygen and Copper adatoms (v_N{3B}−O_{b1-B}−$Cu_{b2-B,B}$) hosted in a 4x4 BN supercell

```
data_v_N-O-Cu_4x4_slab

_cell_length_a 9.98313242
_cell_length_b 9.98322473
_cell_length_c 40
_cell_angle_alpha 90.000000
_cell_angle_beta 90.000000
_cell_angle_gamma 120.943700
_symmetry_space_group_name_H-M       'P 1'
_symmetry_Int_Tables_number          1

loop_
_symmetry_equiv_pos_as_xyz
  'x, y, z'
loop_
_atom_site_label
_atom_site_type_symbol
_atom_site_fract_x
_atom_site_fract_y
_atom_site_fract_z
B001 B 1.937839297143E-01 5.637459056629E-02 0.9740456754772501
Cu002 Cu 7.418484562917E-02 1.756718548036E-01 0.9630643856052499
B003 B 1.555035061913E-01 3.337924212647E-01 0.9989467373357501
N004 N 8.010855629886E-02 4.181343306334E-01 1.0059813907144999
B005 B 1.663472242697E-01 -4.146667932371E-01 1.0064241100562499
N006 N 8.319074515584E-02 -3.331688219559E-01 1.0065758858509999
B007 B 1.675387734221E-01 -1.686273252809E-01 1.00046777447925
N008 N 8.712417213642E-02 -8.894102076547E-02 0.992689330732
B009 B 4.186120279494E-01 8.243755522387E-02 1.0005127002045
N010 N 3.389366435863E-01 1.628734680714E-01 0.99273630182325
B011 B 4.118198402082E-01 3.266140743154E-01 0.99812469659175
N012 N 3.238864628799E-01 4.082605922819E-01 1.00012224362775
B013 B 4.136275416193E-01 -4.205122023155E-01 1.003074046954
N014 N 3.330581168521E-01 -3.356185446776E-01 1.0048036365545001
B015 B 4.178175729927E-01 -1.678320281315E-01 1.0038749877992499
N016 N 3.356751195273E-01 -8.569218033885E-02 1.0010881693905
B017 B -3.353470780199E-01 8.363920618060E-02 1.006458171298
N018 N -4.168502105882E-01 1.667963560425E-01 1.00662326266275
B019 B -3.368176575434E-01 3.339283607826E-01 1.00597153014375
N020 N -4.207007429314E-01 4.156474860631E-01 1.0038647804705
B021 B -3.340686626481E-01 -4.159233476270E-01 1.00376267540125
N022 N -4.170140480182E-01 -3.330019763340E-01 1.00304076325525
B023 B -3.295066338727E-01 -1.636425063160E-01 1.003083861831
N024 N -4.143944576185E-01 -8.306990377583E-02 1.00482245388175
B025 B -8.383181084229E-02 9.447403446218E-02 0.9989539800164999
N026 N -1.681502580947E-01 1.698748125537E-01 1.0060136069435
B027 B -8.736682824022E-02 3.373514157011E-01 1.0066317661405
N028 N -1.692986176816E-01 4.192888059227E-01 1.007598270033
B029 B -8.393704484631E-02 -4.131990048115E-01 1.00595213756225
N030 N -1.656397308115E-01 -3.292966414146E-01 1.003846771164
B031 B -7.660246215492E-02 -1.618140563800E-01 0.9980991822155
N032 N -1.582811268186E-01 -7.390608636189E-02 1.00012120294775
O033 O 1.692459023879E-01 8.107570313173E-02 0.9423620565855
```

Nitrogen vacancy with Oxygen and Copper adatoms ($v_N\{3B\}$–$O_{b1\text{-}B}$–$Cu_{b2\text{-}B,B}$) hosted in a 5x5 BN supercell

```
data_v_N-O-Cu_5x5_slab

_cell_length_a 12.47893550
_cell_length_b 12.47929896
_cell_length_c 40
_cell_angle_alpha 90.000000
_cell_angle_beta 90.000000
_cell_angle_gamma 120.535900
_symmetry_space_group_name_H-M        'P 1'
_symmetry_Int_Tables_number          1

loop_
_symmetry_equiv_pos_as_xyz
   'x, y, z'

loop_
_atom_site_label
_atom_site_type_symbol
_atom_site_fract_x
_atom_site_fract_y
_atom_site_fract_z
B001 B 1.530834530482E-01 4.554175355608E-02 0.46944746924849995
B002 B 1.234475754447E-01 2.694378426352E-01 0.49431622205175
N003 N 6.189055073818E-02 3.358704886676E-01 0.50165728824
B004 B 1.304516778530E-01 4.691452376483E-01 0.50395535900375
N005 N 6.390833684266E-02 -4.652623788474E-01 0.50598610563675
B006 B 1.314681576985E-01 -3.320172908076E-01 0.504370231962
N007 N 6.503889122552E-02 -2.662753168044E-01 0.502764388677
B008 B 1.321776477988E-01 -1.349999156312E-01 0.49632123289275
N009 N 6.790513180265E-02 -7.118666417575E-02 0.487905103671
B010 B 3.335148618520E-01 6.633569124599E-02 0.49637509425075
N011 N 2.697296436124E-01 1.306239967319E-01 0.48794640561174996
B012 B 3.274566070188E-01 2.612857101790E-01 0.4936556141355
N013 N 2.573338804417E-01 3.278003709032E-01 0.4954828369685
B014 B 3.278808959040E-01 4.636898209474E-01 0.4998581924105
N015 N 2.635132926029E-01 -4.677405994770E-01 0.50260527363125
B016 B 3.313458904033E-01 -3.335003790397E-01 0.50308200117425
N017 N 2.650499214695E-01 -2.670973973837E-01 0.5034320143719999
B018 B 3.320161330883E-01 -1.335064566745E-01 0.501605098965
N019 N 2.664511007403E-01 -6.793293789393E-02 0.49732944697150006
B020 B -4.694805891095E-01 6.703159989102E-02 0.5044683363482501
N021 N 4.647647484759E-01 1.334575977816E-01 0.502873162958
B022 B -4.709978803019E-01 2.668245911865E-01 0.50256252682375
N023 N 4.609706779600E-01 3.318749407587E-01 0.49992901613400004
B024 B -4.708918878687E-01 4.659523101884E-01 0.50110101858425
N025 N 4.623537895643E-01 -4.677489664191E-01 0.50052498956325
B026 B -4.686041919017E-01 -3.328963695939E-01 0.5013072216645
N027 N 4.650550959816E-01 -2.665425041739E-01 0.50241868212175
B028 B -4.679847097410E-01 -1.328387001044E-01 0.50312277153
N029 N 4.656039195831E-01 -6.654626762188E-02 0.5034923973797499
B030 B -2.706282138226E-01 6.805065599346E-02 0.5040343178095
N031 N -3.362233787648E-01 1.345931973971E-01 0.50608760804475
B032 B -2.711052921368E-01 2.682820855631E-01 0.506078303653
```

```
N033 N -3.372561126003E-01 3.347341726933E-01 0.5051296180825
B034 B -2.700056637930E-01 4.685086648831E-01 0.50429969511425
N035 N -3.363792547884E-01 -4.651053151180E-01 0.50262539366675
B036 B -2.674396819597E-01 -3.305982309572E-01 0.5010761013937499
N037 N -3.337507751715E-01 -2.638545331460E-01 0.50052938633625
B038 B -2.651828132732E-01 -1.293833588440E-01 0.49986603325549994
N039 N -3.337349204341E-01 -6.500711790741E-02 0.5026594504774999
B040 B -7.094933957909E-02 7.507729061226E-02 0.49433201758449996
N041 N -1.373453569042E-01 1.366134548681E-01 0.50172066760225
B042 B -7.172847806709E-02 2.702518974724E-01 0.50337075126725
N043 N -1.372532537102E-01 3.357672140334E-01 0.50628783247925
B044 B -6.977796147603E-02 4.696140247662E-01 0.5060235904027499
N045 N -1.362271652719E-01 -4.642364425110E-01 0.50506761834925
B046 B -6.832433864942E-02 -3.305081048058E-01 0.50247665526825
N047 N -1.333650100731E-01 -2.624664562162E-01 0.49986885572524997
B048 B -6.276571668139E-02 -1.289340132400E-01 0.49362434635700003
N049 N -1.292953990474E-01 -5.882528003739E-02 0.49545827174925006
O050 O 1.319940227838E-01 6.680714323398E-02 0.43803575413275003
Cu051 Cu 5.762348119276E-02 1.407122435938E-01 0.46014347826625
```

Nitrogen vacancy with Oxygen and Copper adatoms (v_N{3B}−O_{b1-B}−$Cu_{b2-B,B}$) hosted in a 6x6 BN supercell

```
data_v_N-O-Cu_6x6_slab

_cell_length_a 14.92999736
_cell_length_b 14.91462101
_cell_length_c 40
_cell_angle_alpha 90.000000
_cell_angle_beta 90.000000
_cell_angle_gamma 120.618300
_symmetry_space_group_name_H-M        'P 1'
_symmetry_Int_Tables_number          1

loop_
_symmetry_equiv_pos_as_xyz
   'x, y, z'

loop_
_atom_site_label
_atom_site_type_symbol
_atom_site_fract_x
_atom_site_fract_y
_atom_site_fract_z
B001 B 1.154171813320E-01 4.866408622879E-02 0.47185475257325005
B002 B 9.326318297162E-02 2.067453652066E-01 0.50511881284975
N003 N 5.169502349835E-02 2.762396806630E-01 0.5053802586215
B004 B 1.090438542327E-01 3.877999586187E-01 0.5059792911587501
N005 N 5.441904812878E-02 4.443623651237E-01 0.5056612239565
B006 B 1.108021404989E-01 -4.440449281873E-01 0.5046540686500001
N007 N 5.574591717800E-02 -3.881707777568E-01 0.5038592179785
B008 B 1.116471197412E-01 -2.770919036880E-01 0.5000231098997501
N009 N 5.668229625053E-02 -2.213528087758E-01 0.49852563082900003
```

B010 B 1.112881917390E-01 -1.115431612305E-01 0.49131104738799997
N011 N 5.610191461499E-02 -5.680894954357E-02 0.48697318729649997
B012 B 2.777422135790E-01 5.437190514666E-02 0.48889236968875
N013 N 2.228329830184E-01 1.091026733162E-01 0.48478854734474996
B014 B 2.704636668163E-01 2.149875889935E-01 0.49528376706849997
N015 N 2.086092599311E-01 2.638478651329E-01 0.5028893316885
B016 B 2.696498956422E-01 3.794081158315E-01 0.5046409280937499
N017 N 2.196900496384E-01 4.407560048649E-01 0.50618036638425
B018 B 2.767216682651E-01 -4.467456111530E-01 0.5048771231355
N019 N 2.221836640613E-01 -3.900600863960E-01 0.503815747962
B020 B 2.780835131296E-01 -2.786622154200E-01 0.5003266737377501
N021 N 2.228394974399E-01 -2.233022300681E-01 0.49745474850000004
B022 B 2.780729449149E-01 -1.123158171337E-01 0.49318956571774997
N023 N 2.228739399439E-01 -5.769945297570E-02 0.48869504881575
B024 B 4.441891145344E-01 5.485232294789E-02 0.494455560673
N025 N 3.885463676821E-01 1.098442421101E-01 0.49375899748025
B026 B 4.415100482031E-01 2.207183917939E-01 0.49720213928675
N027 N 3.835370165911E-01 2.738017108685E-01 0.49924390163099996
B028 B 4.401798804926E-01 3.851362895312E-01 0.5028662324025001
N029 N 3.832639240265E-01 4.390591035889E-01 0.50427455909775
B030 B 4.421088831325E-01 -4.475550472301E-01 0.50468343320675
N031 N 3.883069368708E-01 -3.905137745635E-01 0.50417523295525
B032 B 4.451038520240E-01 -2.785077640488E-01 0.5021584790035
N033 N 3.897731337984E-01 -2.231904962637E-01 0.49955589328475
B034 B 4.452639284267E-01 -1.117805716437E-01 0.496879144535
N035 N 3.897992988097E-01 -5.674140697608E-02 0.49396505888925
B036 B -3.886179430000E-01 5.590291632240E-02 0.49629864297
N037 N -4.442731709505E-01 1.110819856382E-01 0.49608235065524997
B038 B -3.906269883780E-01 2.224978513442E-01 0.49822646316925
N039 N -4.465424735595E-01 2.773434448760E-01 0.49947885730975
B040 B -3.909793840119E-01 3.886709476912E-01 0.5026252323057501
N041 N -4.471266405167E-01 4.432580078098E-01 0.5041211051860001
B042 B -3.896064296651E-01 -4.444037822891E-01 0.5053111869185
N043 N -4.448448960416E-01 -3.888934075136E-01 0.50510249426325
B044 B -3.863273665446E-01 -2.758697476744E-01 0.50432228083775
N045 N -4.426795927564E-01 -2.214750352385E-01 0.502448606511
B046 B -3.865173675344E-01 -1.100857360341E-01 0.5000815730102499
N047 N -4.427433615985E-01 -5.555330267189E-02 0.49726316340375
B048 B -2.209435031795E-01 5.731523748633E-02 0.49659336062674997
N049 N -2.767737692903E-01 1.123122166592E-01 0.495945349005
B050 B -2.237776508490E-01 2.237214022149E-01 0.49842467426525
N051 N -2.790840420903E-01 2.787097952614E-01 0.4996526732075
B052 B -2.235842253931E-01 3.901563436076E-01 0.50274561560575
N053 N -2.790962062272E-01 4.452611126546E-01 0.5039700883864999
B054 B -2.224361089731E-01 -4.427375760861E-01 0.5051081563415
N055 N -2.772951967376E-01 -3.867733201164E-01 0.5058813939844999
B056 B -2.191381045247E-01 -2.740732679834E-01 0.50519055204575
N057 N -2.730545535946E-01 -2.169656514752E-01 0.5048372596440001
B058 B -2.134314886156E-01 -1.035834663808E-01 0.5035088004752499
N059 N -2.737039502319E-01 -5.266356025456E-02 0.5002155924812499
B060 B -3.815930472003E-02 7.361042606027E-02 0.5034373994482501
N061 N -1.078027421606E-01 1.152248122353E-01 0.49445476163325
B062 B -5.732957236980E-02 2.262563423919E-01 0.5005522184272501
N063 N -1.127952810843E-01 2.799091513304E-01 0.5007953916904999
B064 B -5.660745229502E-02 3.910559462165E-01 0.5039517848277499
N065 N -1.121176497953E-01 4.459109672665E-01 0.5044157880892499
B066 B -5.570649165089E-02 -4.424038807487E-01 0.5045378483115

```
N067 N -1.108128850660E-01 -3.867435065137E-01 0.5045780688629999
B068 B -5.409457162480E-02 -2.749000632684E-01 0.50201387842275
N069 N -1.075988451265E-01 -2.173617197503E-01 0.50296622695675
B070 B -4.895915208186E-02 -1.048664664633E-01 0.4981883766305
N071 N -9.846949282183E-02 -4.304604972437E-02 0.504188839351
O072 O 8.145707728329E-02 8.357268190731E-02 0.44703818278225
Cu073 Cu -2.747377338040E-02 1.047442843000E-01 0.4541870601734995
```

Multidefect: Copper substituting Nitrogen and Nitrogen vacancy with Oxygen and copper adatoms (Cu_N, $v_N\{3B\}-O_{b1\text{-}B}-Cu_{b2\text{-}B,B}$) hosted in a 5x5 BN supercell

```
data_Cu_N__v_N-O-Cu_5x5_slab

_cell_length_a 12.47893600
_cell_length_b 12.47929900
_cell_length_c 40
_cell_angle_alpha 90.000000
_cell_angle_beta 90.000000
_cell_angle_gamma 120.535900
_symmetry_space_group_name_H-M         'P 1'
_symmetry_Int_Tables_number            1

loop_
_symmetry_equiv_pos_as_xyz
   'x, y, z'

loop_
_atom_site_label
_atom_site_type_symbol
_atom_site_fract_x
_atom_site_fract_y
_atom_site_fract_z
B001 B 1.530830000000E-01 4.554200000000E-02 1.0314117250000001
B002 B 1.234480000000E-01 2.694380000000E-01 1.0562804749999999
N003 N 6.189100000000E-02 3.358700000000E-01 1.0636215249999998
B004 B 1.304520000000E-01 4.691450000000E-01 1.0659196
N005 N 6.390800000000E-02 -4.652620000000E-01 1.0679503499999998
B006 B 1.314680000000E-01 -3.320170000000E-01 1.0663344750000001
N007 N 6.503900000000E-02 -2.662750000000E-01 1.064728625
B008 B 1.321780000000E-01 -1.350000000000E-01 1.058285475
N009 N 6.790500000000E-02 -7.118700000000E-02 1.04986935
B010 B 3.335150000000E-01 6.633600000000E-02 1.05833935
N011 N 2.697300000000E-01 1.306240000000E-01 1.04991065
B012 B 3.274570000000E-01 2.612860000000E-01 1.05561985
Cu013 Cu 2.573340000000E-01 3.278000000000E-01 1.057447075
B014 B 3.278810000000E-01 4.636900000000E-01 1.06182245
N015 N 2.635130000000E-01 -4.677410000000E-01 1.064569525
B016 B 3.313460000000E-01 -3.335000000000E-01 1.06504625
N017 N 2.650500000000E-01 -2.670970000000E-01 1.06539625
B018 B 3.320160000000E-01 -1.335060000000E-01 1.06356935
N019 N 2.664510000000E-01 -6.793300000000E-02 1.0592937
B020 B -4.694810000000E-01 6.703200000000E-02 1.0664325749999999
N021 N 4.647650000000E-01 1.334580000000E-01 1.0648374
B022 B -4.709980000000E-01 2.668250000000E-01 1.064526775
N023 N 4.609710000000E-01 3.318750000000E-01 1.06189325
B024 B -4.708920000000E-01 4.659520000000E-01 1.063065275
```

```
N025 N 4.623540000000E-01 -4.677490000000E-01 1.062489225
B026 B -4.686040000000E-01 -3.328960000000E-01 1.063271475
N027 N 4.650550000000E-01 -2.665430000000E-01 1.0643829249999999
B028 B -4.679850000000E-01 -1.328390000000E-01 1.065087025
N029 N 4.656040000000E-01 -6.654600000000E-02 1.06545665
B030 B -2.706280000000E-01 6.805100000000E-02 1.065998575
N031 N -3.362230000000E-01 1.345930000000E-01 1.06805185
B032 B -2.711050000000E-01 2.682820000000E-01 1.06804255
N033 N -3.372560000000E-01 3.347340000000E-01 1.0670938749999999
B034 B -2.700060000000E-01 4.685090000000E-01 1.06626395
N035 N -3.363790000000E-01 -4.651050000000E-01 1.0645896499999998
B036 B -2.674400000000E-01 -3.305980000000E-01 1.06304035
N037 N -3.337510000000E-01 -2.638550000000E-01 1.0624936249999999
B038 B -2.651830000000E-01 -1.293830000000E-01 1.061830275
N039 N -3.337350000000E-01 -6.500700000000E-02 1.0646236999999998
B040 B -7.094900000000E-02 7.507700000000E-02 1.056296275
N041 N -1.373450000000E-01 1.366130000000E-01 1.063684925
B042 B -7.172800000000E-02 2.702520000000E-01 1.065335
N043 N -1.372530000000E-01 3.357670000000E-01 1.068252075
B044 B -6.977800000000E-02 4.696140000000E-01 1.067987825
N045 N -1.362270000000E-01 -4.642360000000E-01 1.067031875
B046 B -6.832400000000E-02 -3.305080000000E-01 1.0644409
N047 N -1.333650000000E-01 -2.624660000000E-01 1.0618330999999999
B048 B -6.276600000000E-02 -1.289340000000E-01 1.0555885999999999
N049 N -1.292950000000E-01 -5.882500000000E-02 1.057422525
O050 O 1.319940000000E-01 6.680700000000E-02 1.0
Cu051 Cu 5.762300000000E-02 1.407120000000E-01 1.0221077250000001
```

Multidefect: Nitrogen substituting Boron and Nitrogen vacancy with Oxygen and copper adatoms (N_B, v_N{3B}−O_{b1-B}−$Cu_{b2-B,B}$) hosted in a 5x5 BN supercell

```
data_N_B__v_N-O-Cu_5x5_slab

_cell_length_a 12.47893600
_cell_length_b 12.47929900
_cell_length_c 40
_cell_angle_alpha 90.000000
_cell_angle_beta 90.000000
_cell_angle_gamma 120.535900
_symmetry_space_group_name_H-M         'P 1'
_symmetry_Int_Tables_number            1

loop_
_symmetry_equiv_pos_as_xyz
   'x, y, z'

loop_
_atom_site_label
_atom_site_type_symbol
_atom_site_fract_x
_atom_site_fract_y
_atom_site_fract_z
B001 B 1.530830000000E-01 4.554200000000E-02 1.0314117250000001
N002 N 1.234480000000E-01 2.694380000000E-01 1.0562804749999999
N003 N 6.189100000000E-02 3.358700000000E-01 1.0636215249999998
```

```
B004 B 1.304520000000E-01 4.691450000000E-01 1.0659196
N005 N 6.390800000000E-02 -4.652620000000E-01 1.0679503499999998
B006 B 1.314680000000E-01 -3.320170000000E-01 1.0663344750000001
N007 N 6.503900000000E-02 -2.662750000000E-01 1.064728625
B008 B 1.321780000000E-01 -1.350000000000E-01 1.058285475
N009 N 6.790500000000E-02 -7.118700000000E-02 1.04986935
B010 B 3.335150000000E-01 6.633600000000E-02 1.05833935
N011 N 2.697300000000E-01 1.306240000000E-01 1.04991065
B012 B 3.274570000000E-01 2.612860000000E-01 1.05561985
N013 N 2.573340000000E-01 3.278000000000E-01 1.057447075
B014 B 3.278810000000E-01 4.636900000000E-01 1.06182245
N015 N 2.635130000000E-01 -4.677410000000E-01 1.064569525
B016 B 3.313460000000E-01 -3.335000000000E-01 1.06504625
N017 N 2.650500000000E-01 -2.670970000000E-01 1.06539625
B018 B 3.320160000000E-01 -1.335060000000E-01 1.06356935
N019 N 2.664510000000E-01 -6.793300000000E-02 1.0592937
B020 B -4.694810000000E-01 6.703200000000E-02 1.0664325749999999
N021 N 4.647650000000E-01 1.334580000000E-01 1.0648374
B022 B -4.709980000000E-01 2.668250000000E-01 1.064526775
N023 N 4.609710000000E-01 3.318750000000E-01 1.06189325
B024 B -4.708920000000E-01 4.659520000000E-01 1.063065275
N025 N 4.623540000000E-01 -4.677490000000E-01 1.062489225
B026 B -4.686040000000E-01 -3.328960000000E-01 1.063271475
N027 N 4.650550000000E-01 -2.665430000000E-01 1.0643829249999999
B028 B -4.679850000000E-01 -1.328390000000E-01 1.065087025
N029 N 4.656040000000E-01 -6.654600000000E-02 1.06545665
B030 B -2.706280000000E-01 6.805100000000E-02 1.065998575
N031 N -3.362230000000E-01 1.345930000000E-01 1.06805185
B032 B -2.711050000000E-01 2.682820000000E-01 1.06804255
N033 N -3.372560000000E-01 3.347340000000E-01 1.0670938749999999
B034 B -2.700060000000E-01 4.685090000000E-01 1.06626395
N035 N -3.363790000000E-01 -4.651050000000E-01 1.0645896499999998
B036 B -2.674400000000E-01 -3.305980000000E-01 1.06304035
N037 N -3.337510000000E-01 -2.638550000000E-01 1.0624936249999999
B038 B -2.651830000000E-01 -1.293830000000E-01 1.061830275
N039 N -3.337350000000E-01 -6.500700000000E-02 1.0646236999999998
B040 B -7.094900000000E-02 7.507700000000E-02 1.056296275
N041 N -1.373450000000E-01 1.366130000000E-01 1.063684925
B042 B -7.172800000000E-02 2.702520000000E-01 1.065335
N043 N -1.372530000000E-01 3.357670000000E-01 1.068252075
B044 B -6.977800000000E-02 4.696140000000E-01 1.067987825
N045 N -1.362270000000E-01 -4.642360000000E-01 1.067031875
B046 B -6.832400000000E-02 -3.305080000000E-01 1.0644409
N047 N -1.333650000000E-01 -2.624660000000E-01 1.0618330999999999
B048 B -6.276600000000E-02 -1.289340000000E-01 1.0555885999999999
N049 N -1.292950000000E-01 -5.882500000000E-02 1.057422525
O050 O 1.319940000000E-01 6.680700000000E-02 1.0
Cu051 Cu 5.762300000000E-02 1.407120000000E-01 1.0221077250000001
```

Multidefect: Oxygen substituting Boron and Nitrogen vacancy with Oxygen and copper adatoms (O_B, $v_N\{3B\}-O_{b1\text{-}B}-Cu_{b2\text{-}B,B}$) hosted in a 5x5 BN supercell

```
data_O_B__v_N-O-Cu_5x5_slab

_cell_length_a 12.47893600
```

```
_cell_length_b 12.47929900
_cell_length_c 40
_cell_angle_alpha 90.000000
_cell_angle_beta 90.000000
_cell_angle_gamma 120.535900
_symmetry_space_group_name_H-M         'P 1'
_symmetry_Int_Tables_number            1

loop_
_symmetry_equiv_pos_as_xyz
   'x, y, z'

loop_
_atom_site_label
_atom_site_type_symbol
_atom_site_fract_x
_atom_site_fract_y
_atom_site_fract_z
B001 B 1.530830000000E-01 4.554200000000E-02 1.0314117250000001
O002 O 1.234480000000E-01 2.694380000000E-01 1.0562804749999999
N003 N 6.189100000000E-02 3.358700000000E-01 1.0636215249999998
B004 B 1.304520000000E-01 4.691450000000E-01 1.0659196
N005 N 6.390800000000E-02 -4.652620000000E-01 1.0679503499999998
B006 B 1.314680000000E-01 -3.320170000000E-01 1.0663344750000001
N007 N 6.503900000000E-02 -2.662750000000E-01 1.064728625
B008 B 1.321780000000E-01 -1.350000000000E-01 1.058285475
N009 N 6.790500000000E-02 -7.118700000000E-02 1.04986935
B010 B 3.335150000000E-01 6.633600000000E-02 1.05833935
N011 N 2.697300000000E-01 1.306240000000E-01 1.04991065
B012 B 3.274570000000E-01 2.612860000000E-01 1.05561985
N013 N 2.573340000000E-01 3.278000000000E-01 1.057447075
B014 B 3.278810000000E-01 4.636900000000E-01 1.06182245
N015 N 2.635130000000E-01 -4.677410000000E-01 1.064569525
B016 B 3.313460000000E-01 -3.335000000000E-01 1.06504625
N017 N 2.650500000000E-01 -2.670970000000E-01 1.06539625
B018 B 3.320160000000E-01 -1.335060000000E-01 1.06356935
N019 N 2.664510000000E-01 -6.793300000000E-02 1.0592937
B020 B -4.694810000000E-01 6.703200000000E-02 1.0664325749999999
N021 N 4.647650000000E-01 1.334580000000E-01 1.0648374
B022 B -4.709980000000E-01 2.668250000000E-01 1.064526775
N023 N 4.609710000000E-01 3.318750000000E-01 1.06189325
B024 B -4.708920000000E-01 4.659520000000E-01 1.063065275
N025 N 4.623540000000E-01 -4.677490000000E-01 1.062489225
B026 B -4.686040000000E-01 -3.328960000000E-01 1.063271475
N027 N 4.650550000000E-01 -2.665430000000E-01 1.0643829249999999
B028 B -4.679850000000E-01 -1.328390000000E-01 1.065087025
N029 N 4.656040000000E-01 -6.654600000000E-02 1.06545665
B030 B -2.706280000000E-01 6.805100000000E-02 1.065998575
N031 N -3.362230000000E-01 1.345930000000E-01 1.06805185
B032 B -2.711050000000E-01 2.682820000000E-01 1.06804255
N033 N -3.372560000000E-01 3.347340000000E-01 1.0670938749999999
B034 B -2.700060000000E-01 4.685090000000E-01 1.06626395
N035 N -3.363790000000E-01 -4.651050000000E-01 1.0645896499999998
B036 B -2.674400000000E-01 -3.305980000000E-01 1.06304035
N037 N -3.337510000000E-01 -2.638550000000E-01 1.0624936249999999
B038 B -2.651830000000E-01 -1.293830000000E-01 1.061830275
N039 N -3.337350000000E-01 -6.500700000000E-02 1.0646236999999998
```

B040 B -7.094900000000E-02 7.507700000000E-02 1.056296275
N041 N -1.373450000000E-01 1.366130000000E-01 1.063684925
B042 B -7.172800000000E-02 2.702520000000E-01 1.065335
N043 N -1.372530000000E-01 3.357670000000E-01 1.068252075
B044 B -6.977800000000E-02 4.696140000000E-01 1.067987825
N045 N -1.362270000000E-01 -4.642360000000E-01 1.067031875
B046 B -6.832400000000E-02 -3.305080000000E-01 1.0644409
N047 N -1.333650000000E-01 -2.624660000000E-01 1.0618330999999999
B048 B -6.276600000000E-02 -1.289340000000E-01 1.0555885999999999
N049 N -1.292950000000E-01 -5.882500000000E-02 1.057422525
O050 O 1.319940000000E-01 6.680700000000E-02 1.0
Cu051 Cu 5.762300000000E-02 1.407120000000E-01 1.0221077250000001
